# Maritime sector pathways toward net-zero emissions within global energy scenarios

**DOI:** 10.1038/s41598-026-35909-4

**Published:** 2026-02-11

**Authors:** Diogo Kramel, Volker Krey, Oliver Fricko, Florian Maczek, Helene Muri, Anders H. Strømman

**Affiliations:** 1 Industrial Ecology Programme (IndEcol), Norwegian University of Science and Technology (NTNU), Trondheim, Norway; 2 International Institute for Applied Systems Analysis (IIASA), Vienna, Austria

**Keywords:** Decarbonization, Shipping, Scenarios, Integrated assessment models, Climate change mitigation, Climate sciences, Energy and society, Environmental sciences, Environmental social sciences, Environmental studies, Scientific community, Social sciences

## Abstract

The maritime sector’s transition toward decarbonization cannot occur in isolation, rather it will be tied to broader transformations in energy, economic, and societal systems. Yet, most existing studies often overlook this integrated perspective, focusing primarily on sector-specific strategies without considering broader societal changes and energy availability on a global scale. To address this gap, this study integrates the MariTeam ship emission model into the MESSAGEix-GLOBIOM integrated assessment framework. Through this approach, we assess how climate scenarios may influence the maritime sector’s trajectory toward achieving net-zero emissions by 2050, in line with the International Maritime Organization (IMO) targets. Our findings indicate that action before 2030 is crucial and it can be achieved through combining four key solutions: improvements in energy efficiency, biofuels, liquefied hydrogen, and ammonia. Furthermore, the results suggest that the maritime sector could have access to enough renewables to achieve substantial emissions reductions with increase in final product costs ranging from 2 to 30% (interquartile range) with variations across products and regions. On average, cost increases are estimated at 10.2% for Global North countries and 13.3% for Global South countries. This analysis highlights the urgency and scale of transformation required for the maritime industry to meet the IMO’s net-zero ambitions and align with broader global sustainability goals.

**Supplementary Information:**

The online version contains supplementary material available at 10.1038/s41598-026-35909-4.

## Introduction

The maritime sector is essential to the global transport network, driving economic development by providing an energy-efficient and cost-effective mode of transportation for international trade^1^. However, the sector’s heavy reliance on fossil fuels has resulted in shipping contributing approximately 2.5% of global CO_2_ emissions annually^2^. As land-based freight transport (i.e., heavy-duty vehicles and rail) reduces emissions through direct electrification, international shipping risks becoming a comparatively less environmentally favorable transport option.

Numerous studies^3–7^ have emphasized that the International Maritime Organization’s (IMO) previous target—to reduce CO_2_ emissions by 50% by 2050—was not aligned with the Paris Agreement. In fact, to be consistent with limiting global warming to below 2 °C, emissions from the sector would need to decline by roughly 88% by 2050^8^. Thus, recognizing the urgency of reducing emissions, the 80th session of the IMO’s Marine Environment Protection Committee (MEPC 80) adopted a revised GHG Strategy. The updated targets call for a 20% reduction in emissions by 2030, a 70% reduction by 2040 (relative to 2008 levels), and the ultimate goal of achieving net-zero emissions “by or around 2050”^9^. This revised goal is substantially more ambitious than its predecessor, especially given the short timeframe for decarbonizing a sector that remains almost entirely dominated by fossil fuels^2^. Moreover, although MEPC 83 has technically approved the principle of a pricing mechanism, the subsequent failure to formally adopt it illustrates the political and economic challenges of enforcing such measures.

Nonetheless, achieving net-zero emissions by 2050 will require a large-scale transition to alternative fuels^10^. The primary fuel candidates for decarbonizing shipping (liquefied natural gas, biofuels, methanol, hydrogen, and ammonia) each have drawbacks. While they can reduce emissions, these fuels generally have lower gravimetric and energy density, which in turn affect storage capacity and sailing range^11^.

Despite recent signs of increased commitment to decarbonization within the maritime sector^12^, structural and operational characteristics make rapid emissions reductions especially difficult. First, the current shipping fleet is highly heterogenous in terms of design, production, and operation (unlike the more serialized production of heavy-duty vehicles, for example)^12^. Furthermore, ships have a relatively long life span (typically around 25 years), resulting in slow fleet turnover and, consequentially, gradual adoption of novel technologies. From a policy standpoint, the industry’s globalized nature and the lack of stringent enforcement mechanisms hinder the implementation of coordinated global strategies in the maritime sector.

On the supply side, substantial efforts are required across the fuel supply chain^13–15^ to make alternative fuels viable, including bunkering infrastructure, refinery readiness, and reliable fuel supply. These challenges create a significant hurdle and perpetuate a “chicken-and-egg” dilemma in which the lack of available alternative fuels inhibits demand, while insufficient demand discourages fuel production and infrastructure investment. This dynamic perpetuates carbon lock-in within the maritime sector^16,17^. Additionally, competition for alternative fuels from other sectors such as aviation^18^, road and rail transport, and various industrial applications, may further constrain fuel availability and drive up costs.

Therefore, the transition of the shipping industry to zero-carbon fuels requires a comprehensive analysis that accounts for the interconnectedness and trade-offs between energy systems, the economy, and the environment. Several studies have explored potential alternatives or decarbonization pathways through life-cycle assessments (LCA)^11,19–22^, sectoral models^23–27^, and economic analyses evaluating the costs of transitioning to cleaner fuels^27,28^. However, the inherent limitations of LCAs can constrain the robustness of conclusions when these assessments are used in isolation.

For example, questions regarding the technical feasibility of certain technologies (e.g., can N_2_O emissions be curbed?^29^); the scalability of specific fuel pathways (e.g., bio-LNG from biowaste is to meet the energy demand of a significant share of the global fleet); implications outside the boundaries of the system (e.g., land-use impacts associated with large-scale biofuel deployment); increase in trade costs; allocation (or competition) of alternative fuels across sectors^30^; the feasibility of achieving transition targets within limited timeframes (e.g., often LCAs are conducted as atemporal analyses).

Similarly, most sectoral models are designed around the goal of decarbonizing the maritime sector in isolation, rather than viewing it as part of the broader global effort to limit warming to 1.5–2 °C. This gap could be addressed by integrating sectoral models with Integrated Assessment Models (IAMs), which are specifically developed to produce coherent, economy-wide decarbonization scenarios. Expanding the system boundaries of studies assessing alternative marine fuels to encompass the entire energy system under different climate trajectories could substantially enhance understanding of the feasible pathways for the maritime sector to achieve the IMO’s net-zero targets.

For a more holistic perspective, IAMs can incorporate a wide range of factors—including the deployment of novel technologies, economic behavior and its effects on trade and shipping, and energy and environmental policies—within one single framework. Because IAMs are integrated with representations of the global economy, they can capture interactions across sectors, such as competition for alternative fuels and model shipping demand as an endogenous variable directly linked to global trade dynamics.

At the same time, the emergence of detailed bottom-up ship emission models, such as STEAM^31^ and MariTeam model^32,33^, provides high-resolution representations of the maritime sector at multiple aggregation levels (e.g., by ship type, region, or route). These models can be coupled to sectoral analysis in IAMs to enhance the accuracy of scenario analysis as demonstrated in this study.

Despite the comprehensiveness of IAMs, relatively few studies have sought to improve the representation of the shipping sector in them. For example, the IMO GHG reports^2,34^ draw on results from IAM scenarios that explore various energy pathways under combinations of Shared Socioeconomic Pathways (SSPs) and Representative Concentration Pathways (RCPs)^35^ to develop long-term shipping demand scenarios. In these projections, ship emissions increase by 5–50% by 2050, largely because the scenarios do not include fuel transition^2^ and the adoption of alternative fuels.

An early effort to link shipping and IAMs was made by Müller-Casseres et al. (2021)^36^, who used the IMAGE model to assess the trade impacts of maritime decarbonization. In their study, shipping fuel demand was endogenized into the IAM, and results showed that meeting a 50% emissions reduction by 2050 would require between 3 and 17 EJ of renewable energy, depending on the scenario. A subsequent multi-model study^8^, involving six global IAMs, expanded this analysis by examining the maritime sector under the “middle-of-the-road” SSP2 scenario while limiting peak warming to 2 °C. The study underscored the importance of drop-in biofuels, renewable alcohols, and green ammonia as key substitutes for conventional fuels to align with global sustainable goals. Meanwhile, results from the Intergovernmental Panel on Climate Change Sixth Assessment Report (IPCC AR6) suggest that shipping would need to fully decarbonize by around 2080 (medium values of IAM scenarios) to align with a > 50% probability of limiting warming to 1.5°C^37^.

However, all these studies predate the adoption of the IMO’s net-zero targets, leading to results in which the sector is still fairly dominated by fossil fuels by 2050 by mid-century. Moreover, none of those studies have incorporated upstream emissions from fuel production that are now explicitly included in IMO’s revised climate ambition.

To address this gap, the present study develops a comprehensive, detailed, and fully assessment of the maritime sector within global decarbonization scenarios. We incorporate the IMO’s latest sectoral targets and assess the implications of achieving net-zero emissions between 2050 and 2070, accounting for both upstream and downstream emissions. This is achieved by coupling a high-resolution ship emission model with the MESSAGEix-GLOBIOM integrated assessment framework. Specifically, we seek to answer three key questions: (i) how do alternative maritime fuel deployment pathways shape future sectoral emissions trajectories? (ii) how does the evolving shipping fuel mix interact with global competition for renewable energy sources? and (iii) how do these dynamics influence final product costs for globally traded commodities?

## Methods

### Interfacing sectoral targets and global scenarios for shipping in IAMs

This study couples the fully bottom-up MariTeam model^32,33^—which combines ship technical specifications, ship position data obtained from satellite data, and weather data in high spatial and temporal resolution to calculate emissions—with the MESSAGEix-GLOBIOM^38–41^ framework, hereafter MESSAGEix, a dynamic systems-optimization modeling framework (specifically, a dynamic recursive equilibrium model with perfect foresight) that enables comprehensive analyses of energy, economy, and the environment in the context of sustainable development and climate change mitigation.

Through this integration, the representation of maritime transport within MESSAGEix is enhanced by incorporating the detailed, high-resolution shipping data generated by MariTeam model, while integrating simultaneously linking the sector to the broader global energy system.

**Fig. 1:**
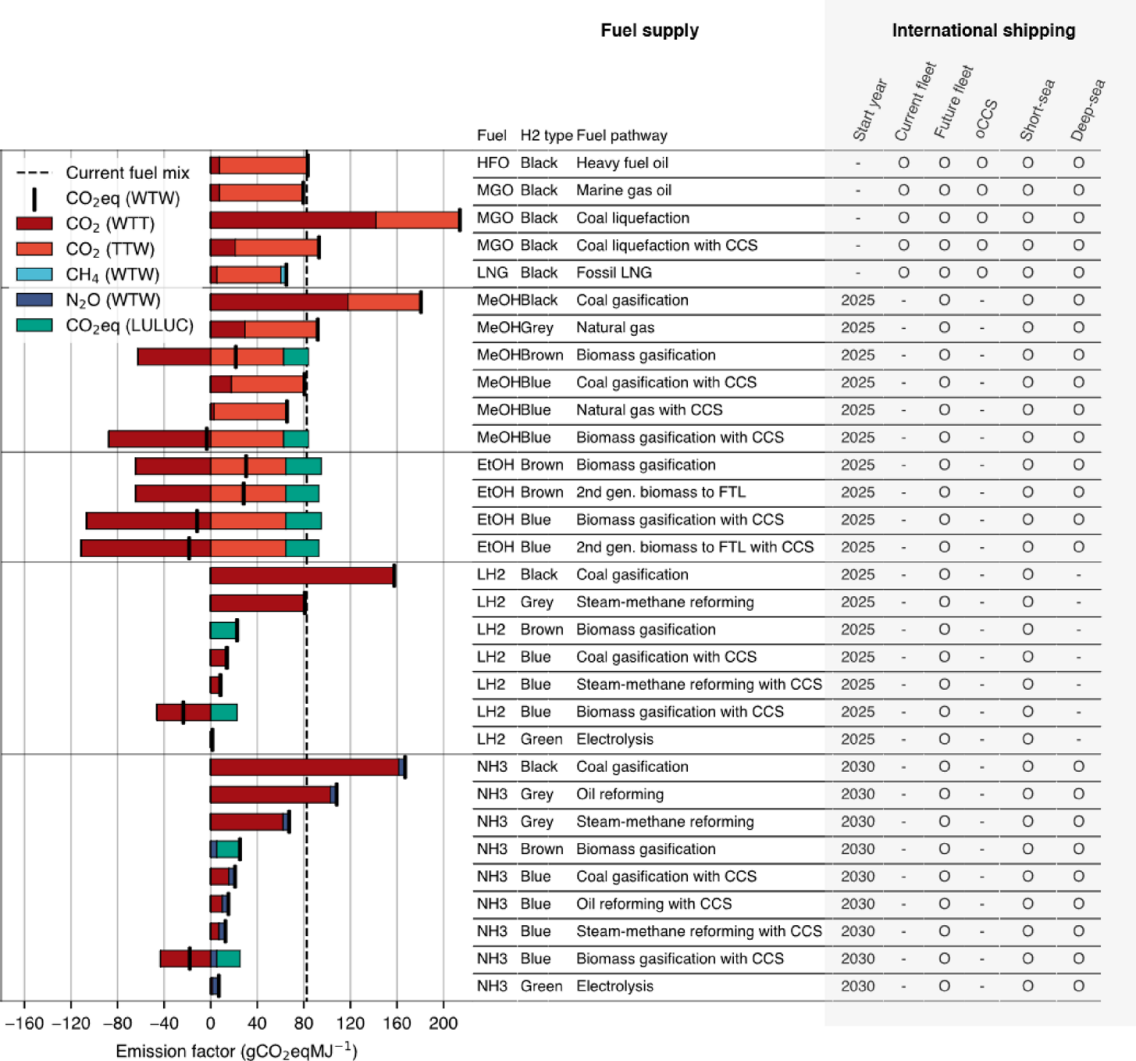
Overview of fuel types, production pathways, associated emissions, and their use in shipping. The bar chart on the left side shows upstream, downstream and LULUC emissions of GHG, as well as resulting net emissions, for each fuel pathway compared to the emission intensity of the current shipping fuel mix. On the right, the fuel supply chain is summarized in how the hydrogen is sourced (black, grey, brown, blue, green) and if the process involves carbon capture and storage. Each fuel pathway, depending on technical constraints, can be used by either the current fleet or newly built ships, with or without OCCS.

The linkage between MariTeam and MESSAGEix is implemented as a soft, unidirectional coupling: energy demand trajectories estimated by MariTeam serve as inputs to MESSAGEix, whereas fuel prices, system costs, and biomass emissions intensities do not feed back into MariTeam. This approach ensures consistency on the energy system’s supply side, although macro-level energy prices (e.g., higher fuel prices in high-cost scenarios) do not endogenously influence modeled shipping activity. A flowchart illustrating the iteration between two models can be found in the Supplementary Methods in the Supporting Information (Fig. 1).

The next sections will detail the processing of shipping energy demand data to make it compatible with MESSAGEix, the linkages built within MESSAGEix to model shipping, and the scenarios that have been analyzed.

### Shipping baseline representation: the mariteam model

The MariTeam model is used to inform the energy demand of international shipping. The model is a bottom-up ship emission model that estimates energy and fuel demand across the global merchant fleet. Fuel demand data is calculate for approximately 50 thousand ships in the tear 2019, out of the roughly 52 thousand merchant ships registered by IMO in the same year^2^. This represents about 97% of the global merchant fleet. The data are then aggregated into seven major ship types (i.e., bulk carriers, car carriers, chemical tankers, container ships, general cargo ships, liquefied natural gas carriers, oil tankers).

Ship types not directly related to international trade (i.e., passenger ships, offshore supply vessels, refrigerated cargo ships) are grouped into a single residual category. The total energy demand represented by MariTeam for 2019 amounts to 9.8EJ, which is consistent with estimates reported in the 4th IMO GHG study—in terms of CO_2_ emissions, the MariTeam model estimates are 9.6% lower than the 4th IMO GHG. For a more extensive validation of the shipping data provided by the MariTeam model, see the section Supplementary Methods in the Supporting Information (SI) and the previous works by the authors^32,33^.

In addition, the MariTeam model includes voyage-level distance data for each vessel, enabling the disaggregation of total energy demand into short- and long-haul voyages (longer than 1000 km). This feature allows the model to capture technological constraints related to fuel range limitations, for instance, the restricted operational range of liquefied hydrogen (LH_2_) vessels.

### Shipping energy demand scenarios

The baseline energy demand estimated by the MariTeam model for the year 2019 is used to develop shipping energy demand projections for the period 2025–2100. To generate these projections, we apply a gravity model of bilateral trade (initially formulated by Tinbergen^42^, inspired by Newton’s law of universal gravitation) to investigate trade flows between country pairs under changing circumstances based on the economic size and the economic barriers between two regions,

In this study, we adopt the same gravity model formulation as developed and described in detail by Kramel et al. (2024)^43^. The model is calibrated for trade data spanning from 1997 to 2023. Approximately 5300 commodities from the CEPII bilateral trade data are mapped to one of seven ship types for each of which a separate gravity model is calibrated. The explanatory variables include GDP and population, which are two key drivers of trade, along with governance, urbanization, income inequality (Gini coefficient), and indicators of whether countries share borders or a common language. After calibration, projections for these variables consistent with the SSP2 are applied, using country-level data from the SSP Extension Explorer^44^. Further methodological details can be found in Kramel et al. (2024)^43^ and the “Supplementary Methods” in the Supporting Information of this study.

In addition, projected fuel demand for each ship type is disaggregated into new builds (ships constructed after 2025) and the current fleet (ships built prior to 2025) using a dynamic stock model^45^ and fleet data covering approximately 50 thousand operating merchant ships. A ship lifetime of 25 years is adopted, implying that most of the current fleet will be fully replaced by around 2050.

The modelling framework described above provides MESSAGEix with the projected fuel demand for ships transporting non-energy commodities. For bulk carriers (transporting biomass, coal, and steel), oil tankers, gas carriers, and chemical tankers (carrying methanol, ethanol, ammonia, and petrochemicals), their fuel demand is adjusted to the demand of the correspondent cargo in MESSAGEix For energy carriers, we calculate the following trade elasticities between energy demand. Elasticity is given as EJ-year of trade per EJ-year of shipping energy demand. Meaning that an elasticity of 0.1 would imply in 10EJ of shipping energy demand for each 100EJ of cargo transported. Results are shown in Table 1.

**Table 1:** Elasticity between trade of commodities and energy demand from the correspondent ship type.

Commodity	Elasticity (EJ/EJ)	Ship type	Percentage of sector transporting the referred commodity in 2020
Crude oil	0.016	Oil tankers	82%
Light oil	0.012	Oil tankers	18%
Liquefied gas (LNG)	0.018	LNG carriers	100%
Coal	0.024	Bulk carriers	25%
Steel	0.059	Bulk carriers	10%
Biomass	0.042	Bulk carriers	0%
Methanol and petrochemicals	0.021	Chemical tankers	100%

### Shipping technology scenarios

The role of technological and operational measures is analyzed in parallel with fuel switching as a means of enhancing overall energy efficiency in the maritime sector and thereby reducing emissions. Three categories of measures are included (i.e., hull design, power & propulsion, operational measures), encompassing to eight specific strategies (i.e., hull shape, air lubrication, hull coating, power system, propulsion system, onboard generation, voyage and speed optimization) that can offer energy efficiency gains between 1 and 8%^46^. When combined simultaneously they could achieve an overall efficiency gain of around 25%. For more details, see Supplementary Note 1 in SI.

This estimate aligns with the range of 5–40% reported in the IPCC Sixth Assessment Report (WGIII, Chap. 10, Fig. 10.15) as potential energy efficiency gains in shipping. It is also consistent with the 4th IMO GHG study, which projects efficiency improvements of 26%, 24% and 25% for bulk carriers, oil tankers and container ships, respectively. Since most energy efficiency measures cannot be retrofitted to existing vessels, they are applied only to future ship cohorts in the model, reflecting their gradual adoption over time. Increases in operational cost are not included, as the selected measures are assumed to have neutral or negative Marginal Abatement Cost (MAC)^2,47,48^.

In addition, onboard carbon capture and storage (OCCS) technologies can be deployed to directly mitigate emissions from diesel and LNG engines^49^. These systems are modelled as mono-ethanolamine (MEA) post-combustion capture systems with flue gas heat integration for diesel and LNG-fueled engines, utilizing heat from engine’s exhaust gases which reduces the fuel penalty to 12% for diesel engines and 9% for LNG engines^49^, the latter benefiting from higher available heat exhaust. Carbon capture rates are considered to be 70% for diesel engines and 85% for LNG engines^49^. Accounting for the fuel penalty, this corresponds to overall emission abatements of 66% for diesel engines and 84% for LNG engines. Reduction in cargo capacity due to OCCS has not been included. From 2030 onward, heavy fuel oil (HFO) engines are assumed to operate with sulfur scrubbers to address air pollution and health concerns related to high sulfur and black carbon emissions. This adds an additional 5% fuel penalty.

Capital and operational costs of OCCS systems are derived from DNV’s ship case study of a China-Europe^50^, expressed in 2020 prices, and correspond to approximately US$36–40 per tonne of fuel. Captured CO_2_ is subsequently transferred back into the MESSAGEix system, where it can either be geologically stored or utilized as feedstock for e-fuel production.

### Enhancing the representation of shipping in MESSAGEix

In MESSAGEix, conventional fuels (i.e., heavy fuel oil—HFO, marine gas oil—MGO, liquefied natural gas—LNG) and alternative fuels (ethanol, methanol, liquefied hydrogen—LH_2_, ammonia—NH3) are implemented to supply the maritime sector. These fuels can be equally supplied to any ship type, meaning that oil tankers and LNG carriers may also operation on alternative fuels if necessary.

Due to the limited onboard storage capacity for fuel tanks, LH_2_ restricted to voyages shorter than 1000 km. Compressed hydrogen is not included in the analysis because of its relatively low volumetric energy density. Regarding propulsion technologies, the model considers only internal combustion engines (ICE) rather than fuel cells (FC), as ICEs are expected to remain the primary power systems for maritime applications in the foreseeable future^51^.

The technology readiness level (TRL) of different fuels and technologies are incorporated into the modeling framework, constraining their earliest possible deployment years. Biofuels and OCCS are available from 2025, whereas ammonia and hydrogen can enter the system in 2030, following potential delays in regulations and the development of port infrastructure. Nuclear energy is not considered in this analysis as nuclear propulsion for commercial shipping remains technologically immature and subject to unresolved regulatory frameworks to facilitate its deployment.

Each fuel is associated with 10 emission species: carbon dioxide (CO_2_), methane (CH_4_), nitrous oxide (N_2_O), volatile organic compounds (VOCs), nitrogen oxides (NOx), carbon monoxide (CO), black carbon (BC), ammonia (NH3), sulfur dioxide (SO_2_), and organic carbon (OC), based on Schwartzkop et al. (2024)^52^. Emission factors are illustrated in Supplementary Notes 2 in SI. Direct ship emissions from the literature are shown in Fig. 1 as Tank-To-Wake (TTW), whereas upstream emissions from fuel production embedded in MESSAGEix are represented as Well-To-Tank (WTT). This structure allows the model to track well-to-wake (WTW), consistent with the net-zero targets established by the IMO. A summary of fuel pathways and associated GHG emissions is shown in Fig. 1.

For ammonia engines, although current N_2_O emissions are relatively high, technological advancements could be able to drastically reduce N_2_O emissions by mid-century, as suggested by novel articles that have achieved GHG reductions of 84%^29^ in ammonia engines. Thus, we model N_2_O emissions declining from approximately 0.778 g/kWh in 2020 and falling to 0.015 g/kWh in 2050, following Schwarzkopf et al. (2023)^52^. Their study compared an uncontrolled ammonia engine technology (compression ignition engine with marine gas oil as pilot fuel) versus controlled technology (a spark ignition engine using hydrogen as the pilot fuel and exhaust gas treatment).

Land-use and land-use-change (LULUC) are also explicitly considered, as the large-scale bioenergy production required under 1.5 °C and 2 °C scenarios carries significant associated emissions. In MESSAGEix-GLOBIOM, bioenergy supply is derived from GLOBIOM land-use modelling, which explicitly represents energy crops (e.g. miscanthus, switchgrass, short-rotation coppice), forestry biomass, and agricultural residues^38,53^. These feedstocks are aggregated into regional biomass supply curves that feed into MESSAGEix.

Because of the integrated nature of this modeling approach, assessing the emissions associated with bioenergy production for a specific sector (i.e. shipping) is not straightforward, even though GLOBIOM explicitly represents energy crops. Thus, to derive emission factors that are internally consistent with the MESSAGEix-GLOBIOM framework, this study uses the same procedure as adopted by the IPCC AR6^54^ to derive emissions associated with primary biomass supply using stylized scenarios from EMF-33^55^. In this method, land-use emissions are calculated as the difference between a baseline scenario and a counterfactual scenario with no bioenergy demand. The cumulative land-use emissions between 2020 and 2100 are divided by cumulative bioenergy production over the same period, yielding an average emission factor of 19gCO2eqMJ^− 1^.

### Global scenarios

**Fig. 2:**
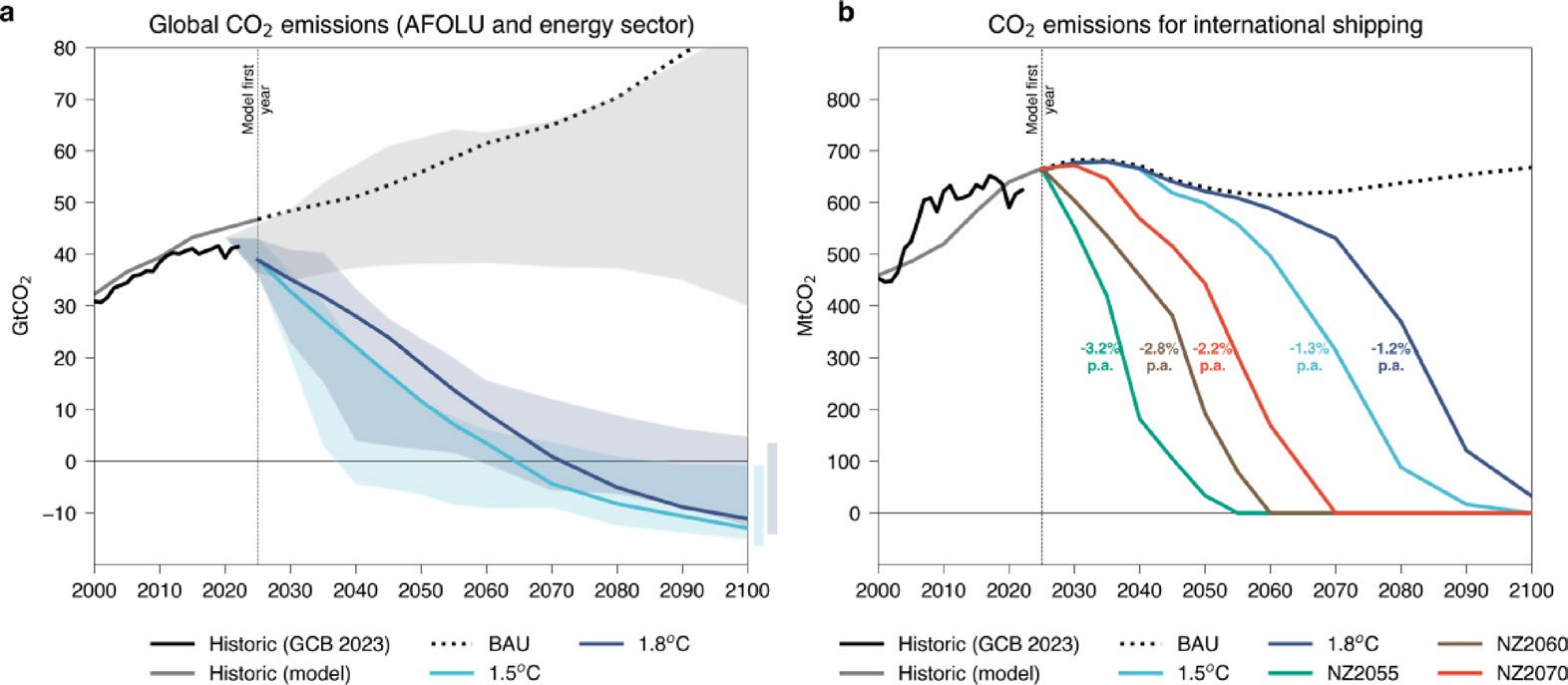
Emission pathways for global CO_2_ emissions and shipping CO_2_ emissions. Plot (**a**) shows global CO_2_ emissions for a business-as-usual (BAU) scenario compared to the 1.5 °C and 1.8 °C scenarios, which are contrasted with C3 and C4 scenarios available in the IIASA scenario database and the historic emissions from the Global Carbon Budget^56^ (GCB). Plot (**b**) shows shipping emission trajectories for the same scenarios, in addition to scenarios with sectorial target to reach net-zero emissions by 2055, 2060 and 2070 (NZ2055, NZ2060, and NZ2070) with their average annual emission reduction rates.

Two sets of scenarios representing illustrative global mitigation pathways are analyzed in this study (hereafter referred to as G1.5 °C and G1.8 °C). The G1.5 °C scenarios have around 600GtCO_2_ of cumulative emissions until net-zero and 300GtCO_2_ for the period 2021–2100. They are equivalent to IPCC C2 scenarios (IPCC, 2022) (1.5 °C with high overshoot) that limits warming to around 1.5 °C, with global net-zero CO₂ emissions reached around 2060. The second variant, namely G1.8 °C scenarios, has 1000GtCO_2_ of cumulative emissions until net-zero and 800GtCO_2_ of cumulative emissions until 2100, corresponding to the IPPC C3 scenarios (IPCC, 2022) (likely below 2 °C), where warming peaks at 1.8 °C throughout the 21st century reaching net-zero emissions globally around 2070 (see Fig. 2a). To align the system with these mitigation trajectories, carbon price signals of US$191 tCO_2_^–1^ (for the 1.5 °C case) and US$102 tCO₂⁻¹ (for the 1.8 °C case) are introduced from 2025 onward. These two scenario sets were chosen to explore distinct climate mitigation pathways with varying levels of cumulative emissions and net-zero timing, enabling a comparative analysis of the implications of different warming trajectories on the shipping sector and broader decarbonization strategies.

For the shipping sector, sectoral variants are developed within both G1.5 °C and G1.8 °C scenarios, pushing the sector to reach net-zero emissions “by or around 2050” as proposed in the IMO’s revised GHG strategy. In this study, this corresponds to no later than 2055 due to MESSAGEix running on 5-year time resolution, as well as 2060 and 2070 if the sector fails to reach the target on time (see Fig. 2b). This allows us to investigate the attainability of sectoral targets and the implications in the shipping fuel composition (scenarios 3, 4, 5, 7, 8, and 9 in Table 2) under delayed net-zero pathways. Regional or domestic regulatory instruments (e.g. EU ETS, FuelEU Maritime, UK ETS extensions) are not included because integrating these heterogeneous multi-jurisdictional policies into the global optimization framework is non-trivial and would likely require a separate modelling effort.

Additionally, a sensitivity analysis is performed in which the deployment of certain fuels is constrained (i.e., ammonia, biofuels), resources (i.e., biomass for fuel production), or technology (i.e., limiting energy efficiency gains) in the baseline scenarios (scenarios 10 to 15 in Table 2). This way, we can explore technological uncertainties regarding the deployment of alternative fuels within the timeframe of the IMO’s net-zero goal across the 15 scenarios.

**Table 2:** Main scenarios included in this study summarizing key scenario characteristics, including peak global warming and cumulative CO_2_ emissions during this century, the years in which the world and the shipping sector each reach net-zero emissions, the accounting scope of emissions (upstream and downstream), and any deployment constraints applied to fuels or technologies.

	Scenario name	Peak warming target	Global budget (GtCO2)	World net-zero year	Shipping net-zero year	Shipping emissions accounting	Fuels and technology availability
1	BAU	–	–	–	–	–	All
2	G1.8^o^C	1.8 °C	1000	2075	–	–	All
3	NZ2055-WTW-1.8^o^C	1.8 °C	1000	2075	2055	WTW	All
4	NZ2060-WTW-1.8^o^C	1.8 °C	1000	2075	2060	WTW	All
5	NZ2070-WTW-1.8^o^C	1.8 °C	1000	2075	2070	WTW	All
6	G1.5^o^C	1.5 °C	600	2065	-	-	All
7	NZ2055-WTW-1.5^o^C	1.5 °C	600	2065	2055	WTW	All
8	NZ2060-WTW-1.5^o^C	1.5 °C	600	2065	2060	WTW	All
9	NZ2070-WTW-1.5^o^C	1.5 °C	600	2065	2070	WTW	All
10	NZ2055-WTW-1.5^o^C-NONH3	1.5 °C	600	2075	2055	WTW	No ammonia fuel
11	NZ2055-WTW-1.5^o^C-NOBIOF	1.5 °C	600	2075	2055	WTW	No biofuels
12	NZ2055-WTW-1.5^o^C-NOBIOM	1.5 °C	600	2075	2055	WTW	No biomass-based fuels
13	NZ2055-WTW-1.5^o^C-NOCCS	1.5 °C	600	2075	2055	WTW	No upstream CCS
14	NZ2055-WTW-1.5^o^C-NOEFF	1.5 °C	600	2075	2055	WTW	No energy efficiency
15	NZ2055-WTW-1.5^o^C-OILGAS	1.5 °C	600	2075	2055	WTW	Limit oil and gas tankers

## Results

### Shipping fuel transition toward mid and end of the century

In all scenarios, shipping energy demand increases until 2050 and stabilizes toward the end of the century peaking at 16EJyr^− 1^ for the G1.5 °C scenarios and 17.5EJyr^− 1^ for the G1.8 °C scenarios. In both cases, energy efficiency improvements are vital for reducing 26% of total energy demand down to 11.3 and 12.4EJyr^− 1^, respectively. The remainder of the energy demand is supplied through different fuel sources.

**Fig. 3:**
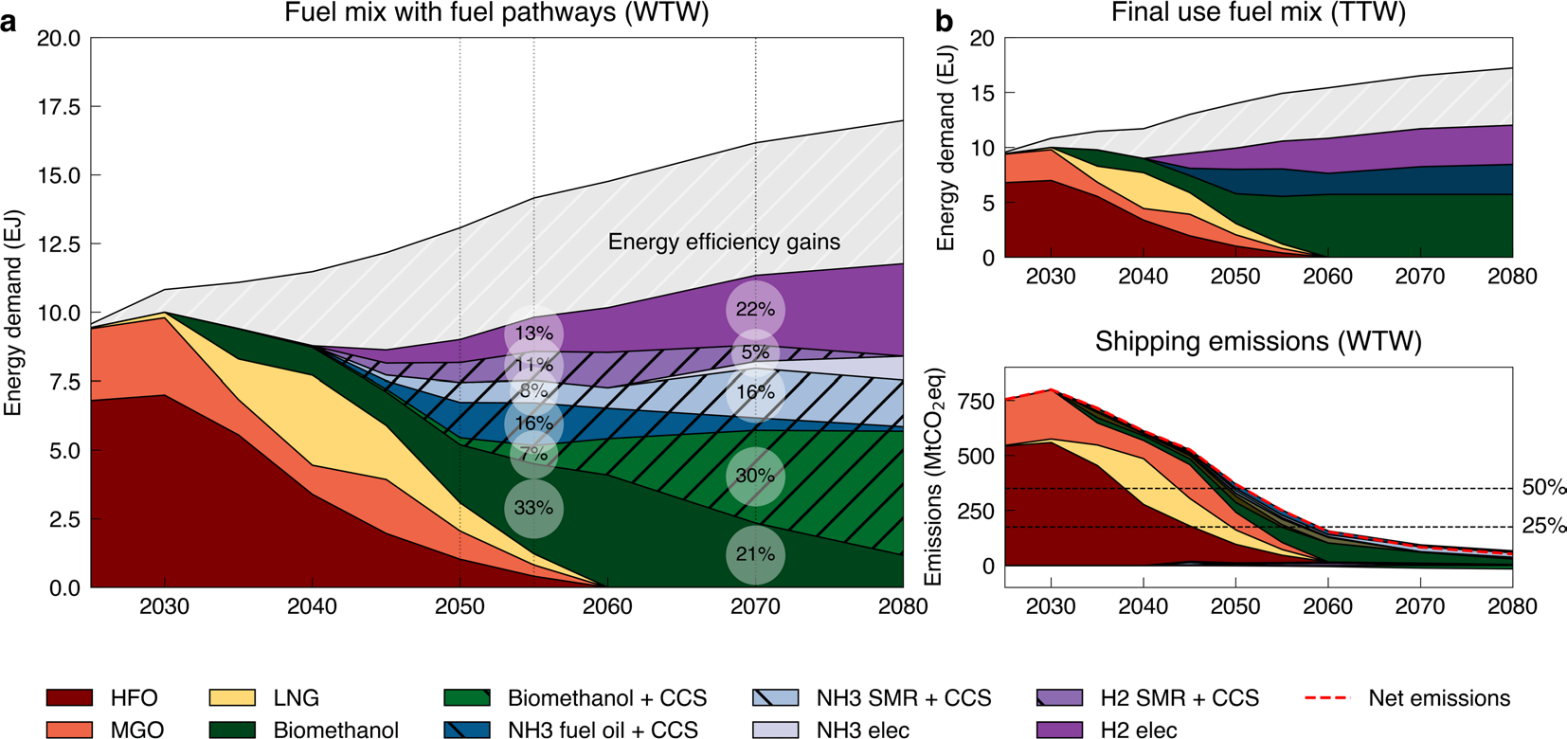
Fuel pathways for different transition scenarios. Figure (**a**) shows the fuel pathways resulting from a scenario in which shipping reaches net-zero emissions by 2055. Figure (**b**) shows the fuel available for the fleet (top) and the emissions associated with the fuels (bottom), negative representing OCCS in the case of fossils or BECCS in the case of methanol.

Figure 3 illustrates results for the scenario investigating the shipping sector reaching the IMO target of net-zero emissions by 2055 (NZ2055-WTW-1.5 C). Results indicate three distinct phases in shipping’s transition to greener fuels.

**Phase 1 — Fossil fuel phase-out (2020–2050): **The gradual replacement of heavy fuel oil (HFO) and marine gas oil (MGO) is supported by the temporary adoption of liquefied natural gas (LNG) as a transition fuel. During this phase, onboard carbon capture and storage (OCCS) is not deployed to offset emissions.

**Phase 2 — Transition to blue ammonia and BECCS (2050–2070): **Beginning around 2040, the sector increasingly relies on blue ammonia (produced via steam–methane reforming with CCS) alongside bioenergy with carbon capture and storage (BECCS), which provides net-negative GHG emissions. By 2055, when the sector reaches net-zero emissions, the fuel mix consists of approximately 40% ammonia, 40% biofuels, and 20% energy efficiency gains.

**Phase 3 — Expansion of green fuels (post-2070): **After 2070, green fuels produced from electrolysis (ammonia and hydrogen) become available and cost-competitive for for shipping, alongside increasing deployment of bio-methanol with upstream CCS.

**Fig. 4:**
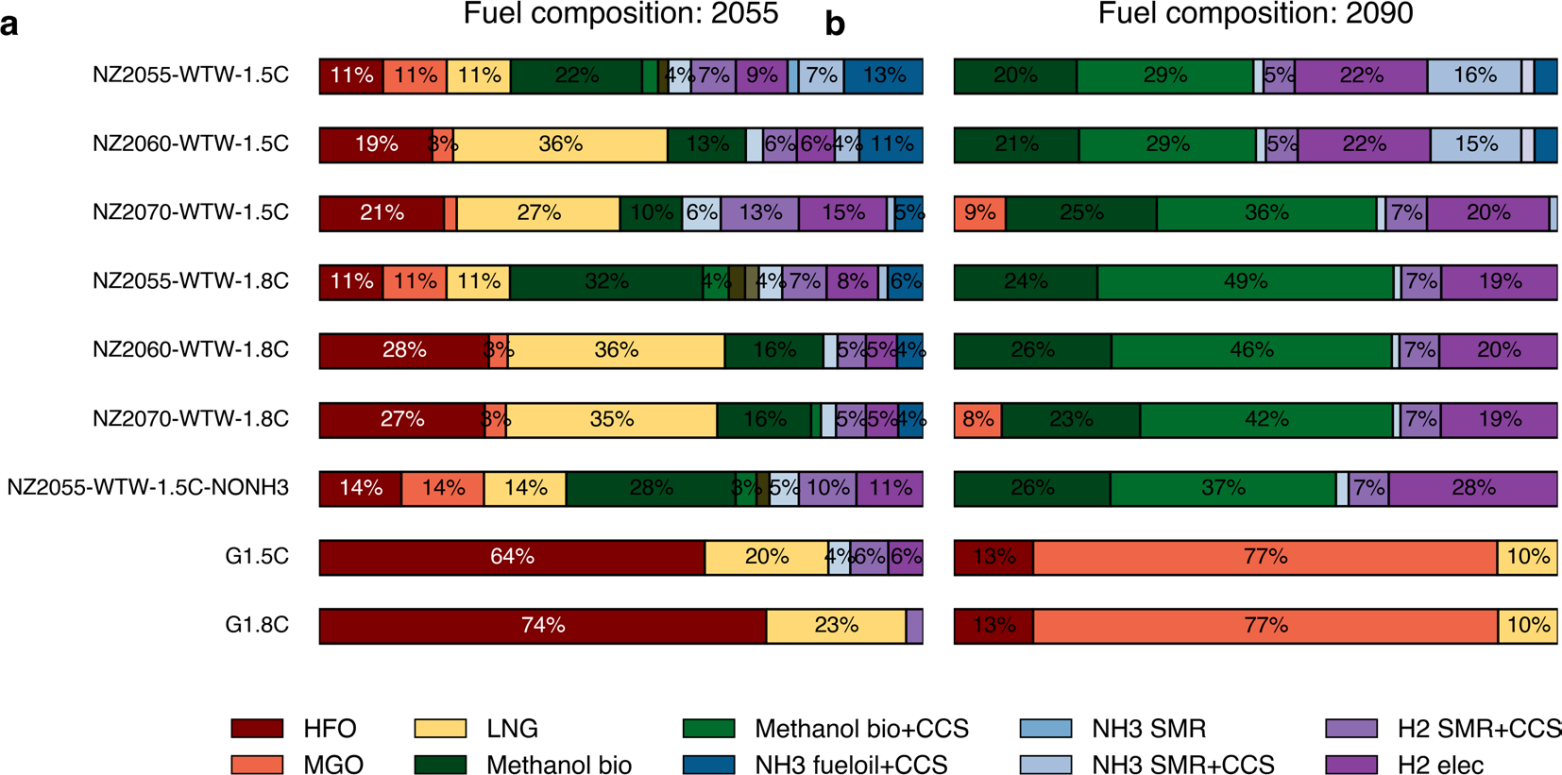
Fuel composition of the global shipping fleet in 2050 and 2090 across all scenarios that achieved feasibility under the constraints listed in Table 2.

If the achievement of net-zero emissions is delayed by 10 or 20 years (NZ2060 and NZ2070 scenarios), the transition follows a similar trajectory but is shifted later in time. For these and other scenarios results for the year 2050 and 2090, see Fig. 4. By the end of the century, the fuel mix converges across all scenarios, dominated by BECCS (net-negative emissions) and ammonia (marginally net-positive emissions). This outcome arises because the model requires a combination of negative and positive emission fuels to balance residual emissions and achieve overall net-zero.

**Fig. 5:**
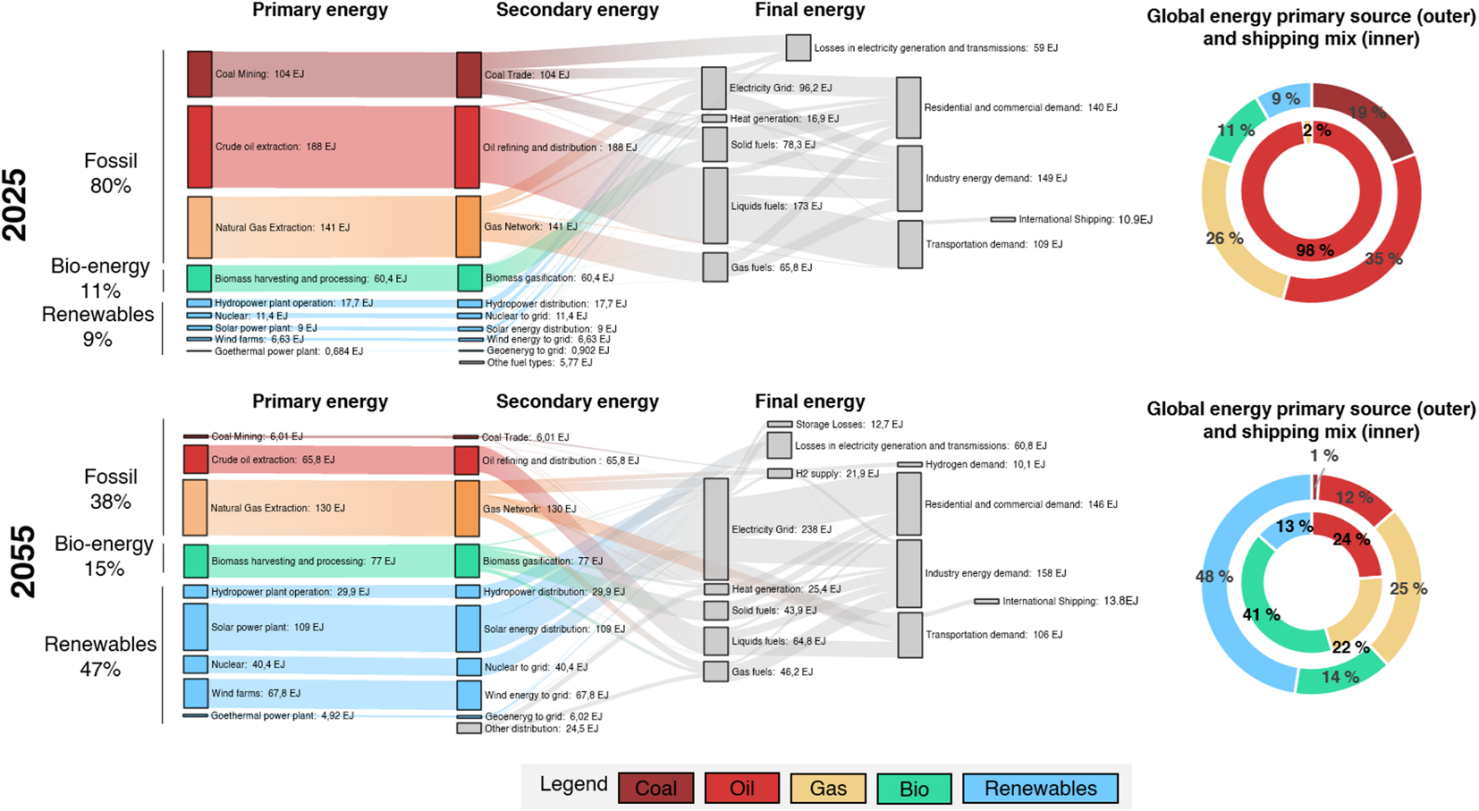
Shipping as part of the global energy transition. Sankey diagrams of flow of energy from extraction to distribution and final energy in the residential, industry and transportation sector for the years 2025 and 2055. Pie charts show the energy mix in the global primary source and the shipping sector.

Alternative scenarios are also explored where specific fuels are unavailable to the sector due to economic, political, or resource constraints. These include the NZ2055-WTW-NOBIOF (no biofuels for shipping), NZ2055-WTW-NOBIOM (no biomass-derived fuels), and NZ2055-WTW-NONH3 (no ammonia). Because only biomass-based fuels with CCS can achieve net-negative emissions, the NOBIOF and NOBIOM scenarios cannot attain net-zero. In the NONH₃ scenario, the sector relies almost entirely on biofuels, with limited use of electrolysis-based fuels (see Fig. 5).

Although the 1.5 °C and 1.8 °C scenarios differ in global mitigation stringency, they lead to remarkably similar decarbonization pathways for the maritime sector, as seen in all NZXX-WTW-X.XC scenarios in Fig. 4. This convergence occurs because the IMO’s ambitious net-zero target compels early and aggressive adoption of low-carbon technologies, leaving little room for variation between scenarios.

Overall, the model framework depends heavily on the scalability of CCS to reduce upstream emissions, particularly for biofuels and ammonia, until green hydrogen and green ammonia from electrolysis becomes viable. When CCS is excluded from the solution space, the model fails to yield feasible outcomes, consistent with the emission factors shown in Fig. 1.

Results from this study differ notably from those of previous works that have used integrated assessment models (IAMs) to explore maritime sector decarbonization pathways. For example, Müller-Casseres et al. (2023)^8^ found that fossil fuels still dominate the shipping fuel mix by 2050, primarily due to the absence of a sector-specific regulatory framework in their modeling approach, which relies solely on a global carbon budget. In contrast, Speizer et al. (2023) projected a strong dominance of hydrogen (around 50% from 2060) in future shipping fuels. However, in our analysis, hydrogen deployment is intentionally constrained to reflect the current technical and operational limitations of using hydrogen at scale in maritime applications, an aspect that was not captured in their model that did not include ammonia as a fuel alternative. Together, these differences highlight the importance of explicitly incorporating both sectoral net-zero targets and technological feasibility constraints (e.g., ammonia’s role) in modeling the maritime transition.

By comparison, DNV’s Maritime Forecast report^50^ shows that a combination of biofuels and ammonia are especially suitable fuels^8,57^ for the shipping sector in the coming decades. The role of LNG as part of the energy transition and not as a definite solution^58^ is seen, but results indicate a very small contribution in reducing near-term emissions. According to DNV’s Maritime Forecast report^50^, low- and zero-carbon fuels are expected to make up approximately 84% of the maritime fuel mix by 2050 (with ammonia at 36%, biofuels at 25%, and e-fuels at 19%). These figures indicate a substantially lower prevalence of ammonia in our results, with biofuels emerging as the most widely deployed fuel option.

E-diesel and e-methanol, in particular, are found to be unattractive for the sector due to their low overall energy efficiency, high production costs, and continued CO₂ emissions during combustion—emissions that would need to be recaptured to avoid net increases in GHGs. Consequently, liquefied hydrogen (LH_2_) and e-ammonia increase relevance toward the end of the century as renewable electricity capacity expands and prices decrease.

Beyond demonstrating the technical feasibility of meeting the IMO’s net-zero goals, the transition to alternative fuels also provides additional environmental co-benefits, including substantial reductions in aerosols, especially black carbon and sulfur dioxide (SO₂), that are not addressed by conventional mitigation measures.

### Availability of renewables for international shipping

Since the shipping sector will not decarbonize in isolation, its energy transition is examined here as part of the broader global energy system to assess what it entails for the sector to achieve net-zero emissions by 2055 in the NZ2055-WTW-1.5 °C scenario. To this end, the shipping energy mix is compared with the global primary energy mix between 2025 (the simulation’s baseline year) and 2050, when the sector approaches net-zero GHG emissions.

As shown in Fig. 5, the global primary energy mix shifts dramatically during this period, going from approximately 80% fossil fuels in 2025 to 38% by 2050, driven by a substantial expansion of renewables (from 9% to 47%) and a moderate increase in bioenergy (from 11% to 15%). In contrast, international shipping represents only a small share of total final energy demand compared to global primary energy production (~ 10 EJ versus 520 EJ, or about 1.9%). Consequently, even ambitious sectoral decarbonization targets in shipping will have only a modest impact on global energy supply. The shipping sector alone will not drive large-scale demand for low-carbon fuels but will instead depend heavily on the broader global energy transition.

The pie charts on the right-hand side of Fig. 5 highlight that, in this scenario, shipping transitions from being a traditionally late-decarbonizing sector to one that is ahead the global energy transition in terms of renewable adoption and fossil fuel phase-out. As a result of the IMO’s revised GHG Strategy, the sector moves from almost entirely dependent on fossil fuels in 2025 to reduce fossil fuel use to roughly 46% by 2055, with a large portion (39%) being ammonia and hydrogen production with upstream CCS.

The 1.5 °C pathways considered here involves a temporary temperature overshoot of up to 1.6–1.8 °C around 2050–2060. Further reducing this overshoot would require more aggressive action across the broader global economy, bringing it more in line with the pace of decarbonization observed in the shipping sector.

### Implications for trade and final product costs

To assess the economic implications of potential decarbonization pathways for the shipping sector, we assess how the increase in fuel costs could impact the final price of traded goods. For that, the increase in bunkering fuel prices (i.e., diesel, LNG, methanol, ethanol, ammonia and LH_2_) in the NZ2055-WTW-1.5 C scenario is compared to the current costs of shipping. Fuel prices peak around 2060, reaching roughly 3.5 times their 2025 levels. Assuming that fuel accounts for approximately half of total operational costs, with the remaining 50% attributed to other constant expenses, this results in an overall increase of 2.25 times in ship operating costs, or a 125% increase. For comparison, DNV’s estimates that costs could increase by 69–112% by 2050^50^. These costs are then combined with the final product cost shares related to shipping alone obtained from UN Comtrade’s Maritime Transport Costs^59^, covering a total of 37 countries for the years 2005, 2006, and 2007.

**Fig. 6:**
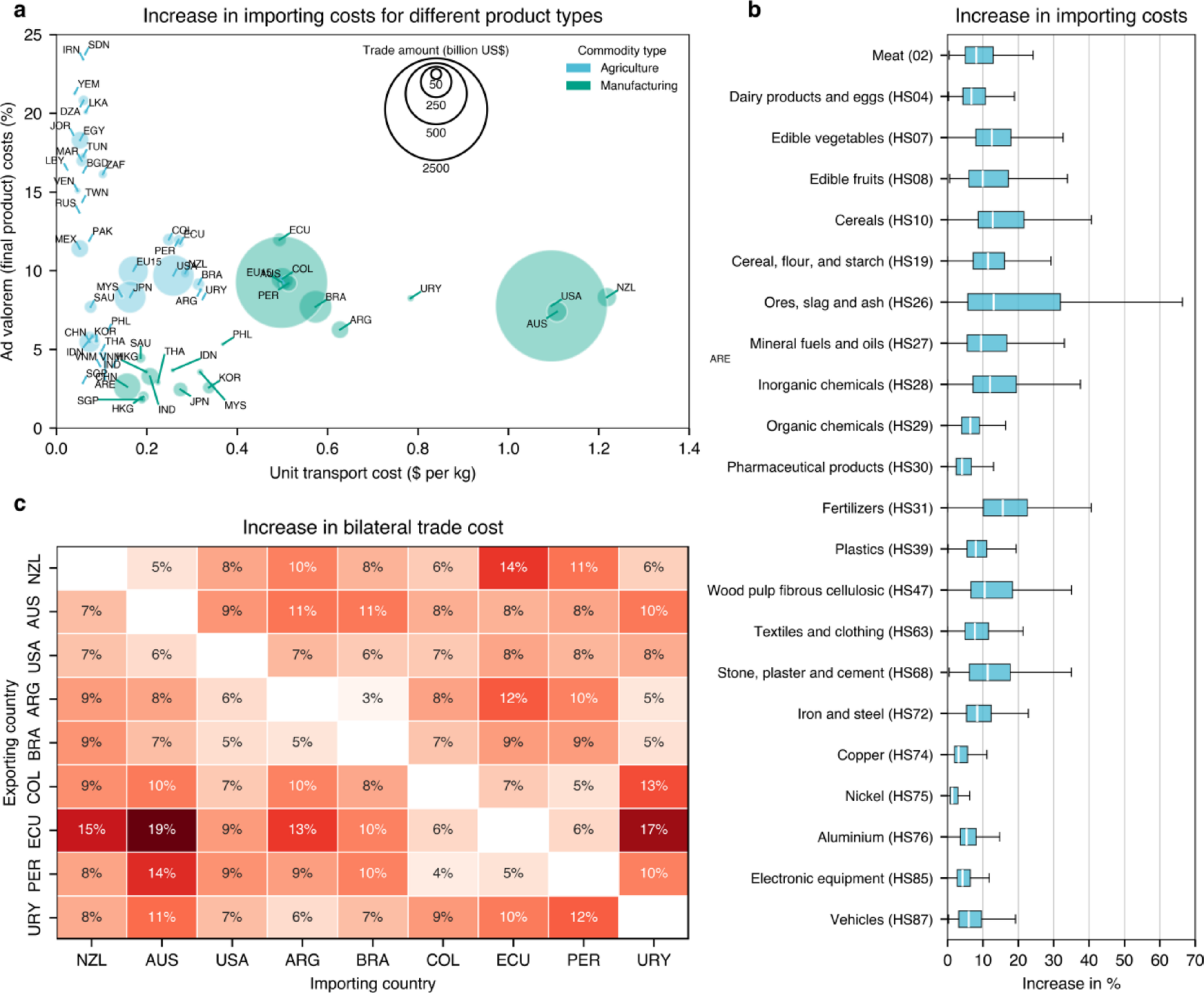
Implications of increased fuel price in products final cost. Figure (**a**) shows the final product costs and unit transport cost increase for different economies for agriculture and manufacturing commodities. Figure (**b**) shows the spread across countries of increase in final product cost for key commodities in the HS system. Figure (**c**) shows the increase in bilateral trade costs for pairs of countries in south America, Oceania and the US.

In Fig. 6a, results show that smaller economies (represented by circle radius) and those in geographically disadvantaged locations are the most affected in terms of final product costs (y-axis) for low added-value agriculture commodities (blue). We also note that manufactured products are comparatively less affected, as transportation costs represent a smaller fraction of their overall market value.

The geographical position is also relevant when assessing the impact of bilateral trade in Fig. 6c. Countries like Australia and Ecuador that are not in close proximity to major shipping routes are the ones most affected, reaching an increase in costs of trade up to 19%. It is important to note that due to asymmetric trade patterns between country pair, values differ between importing and exporting countries.

Figure 6b further illustrates that high–value-added goods (e.g., electronics, pharmaceuticals, and vehicles) are largely insulated from rising fuel costs, with median increases below 5%. In contrast, bulk commodities such as ores, fertilizers, cement, and cereals could see cost increases approaching 15%. The distinction between low- and high–value-added goods is also evident within categories, such as metals (iron and steel versus copper and nickel) and food products (fruits and vegetables versus meat and dairy), emphasizing how decarbonization may disproportionately burden low-margin sectors. It is important to note that the third quartile can be as high as 30%, showing that certain commodities and countries might be severely impacted by the maritime sector’s fuel transition. Results disaggregated by commodity type and region are presented in Supplementary Note 6 of the Supporting Information. On average, cost increases are estimated at 10.2% for Global North countries and 13.1% for Global South countries.

It should be noted, however, that these estimates represent a direct transmission of fuel cost increases to product prices, without accounting for broader macroeconomic feedbacks or adaptive responses in trade, logistics, and technology. In practice, the consumer-level price effect could be in fact smaller, as decarbonization-induced cost pressures are distributed across the global economy. For instance, in the same NZ2055-WTW-1.5 °C scenario, the model projects price increases of 18% for electricity in Eurasia, 27% in Asia, 6% for steel, and 18% for aluminum. Overall, the analysis underscores that while decarbonizing maritime transport may affect high-value global supply chains less severely than low-added products, it risks amplifying cost disparities for resource-dependent and geographically isolated economies, an important consideration for equitable climate policy design.

## Discussion

This study examined the maritime sector’s transition toward net-zero emissions within the broader context of the global energy system by coupling a high-resolution bottom-up ship emission model (MariTeam) with the integrated assessment framework MESSAGEix-GLOBIOM. Through this linkage, we explored how the International Maritime Organization’s (IMO) revised target of achieving net-zero greenhouse gas (GHG) emissions “by or around 2050” could be met under distinct global mitigation pathways.

Our findings demonstrate the importance of representing the shipping sector in greater detail within Integrated Assessment Models (IAMs) to better capture the sector’s interactions with global energy, trade, and economic systems. The soft-coupling approach introduced here allows for improved resolution in shipping energy demand across ship types, routes, and voyage lengths, while maintaining system-wide consistency in energy and emission accounting. This framework provides a foundation for further integration, where shipping demand could eventually be endogenized as a function of trade dynamics, fuel costs, and global economic feedback. As of now, potential demand responses to higher fuel prices or endogenous operational efficiency effects are not fully captured.

Nonetheless, IAM-based approaches inevitably rely on simplifications, particularly regarding technology detail. The representation of alternative fuels, especially biofuels and green hydrogen, remains idealized due to aggregated assumptions on biomass sources and conversion efficiencies. Future studies should therefore complement IAM analyses with life-cycle assessment (LCA) and spatially explicit land-use modeling to evaluate biodiversity implications and regional trade-offs in resource use. Indeed, our scenarios indicate that the scale of bioenergy deployment required at a global level could contribute to significant natural forest losses, approximately 25% and 33% in the 1.5 °C and 1.8 °C pathways, respectively, due to extensive system-wide deployment of Bioenergy with carbon capture and storage (BECCS), underscoring the need for more robust land-use and sustainability constraints in future modeling.

Technological uncertainties also remain a key limitation. Parameters such as the operational range of liquefied hydrogen (LH_2_) ships, the evolution of N₂O emissions from ammonia engines, and the effective loss of cargo space associated with new fuel systems introduce considerable uncertainty into long-term projections. Similarly, assumptions on carbon capture and storage (CCS) deployment (both upstream and onboard) are critical model drivers. The reliance on BECCS and blue ammonia to achieve net-zero outcomes should therefore be interpreted as a structural necessity of the modeling framework rather than a definitive forecast of future technology mixes. Our study has not carried out a qualitative assessment of the main safety challenges of fuels such as ammonia and hydrogen, but these should be considered nonetheless. Ammonia is weakly flammable but highly toxic, while hydrogen is non-toxic but extremely flammable with a very wide ignition range, meaning their safe deployment hinges on different dominant hazards. Furthermore, at scale, the routine release or accidental spillage of ammonia could materially affect marine ecosystems, particularly given its toxicity to marine life, which is not captured in our modelling framework. Besides the risks, the model does not capture the reduction of aerosols and other pollutants that would stem from transitions to cleaner fuels, which could be addressed in future work, as the distribution of these short-lived species could have significant health implications in port cities.

The results reveal that, even under ambitious decarbonization pathways, achieving win–win outcomes that are both economically and environmentally optimal remains unfeasible^60^. Significant trade-offs between cost, scalability, and sustainability are evident across all scenarios. In particular, while hydrogen, ammonia and biofuels emerge as key pillars of the transition, their widespread adoption depends heavily on the pace of global renewable energy expansion and the establishment of large-scale carbon management infrastructure. The shipping sector alone will not be the primary driver of demand for green fuels; rather, it will depend on the broader energy system’s transformation to ensure adequate supply and cost parity with fossil alternatives.

Operationally, the IMO’s net-zero target requires immediate and coordinated action across the entire value chain. An “all-hands-on-deck”^12^ approach is critical to accelerate the deployment of alternative fuels and facilitate their widespread adoption by mid-century aligned with the natural turnover of the fleet providing a critical window to introduce new fuel technologies and vessel designs. Delays in fuel deployment or infrastructure development would lock in higher emissions trajectories and increase the risk of stranded assets or costly retrofits. While onboard carbon capture and storage (OCCS) can act as a bridging measure, its cost and energy penalty limit its long-term role. Besides that, technical aspects (i.e., corrosion, safety, logistics, CO₂ handling and storage) that could hinder the deployment of OCCS have not been modelled.

The economic assessment highlights that the transition to low- and zero-carbon fuels could substantially increase shipping costs, with potential knock-on effects on global trade and commodity prices. However, the burden will not be evenly distributed. High–value-added manufacturing sectors are relatively insulated, as transportation represents a small share of their market price, whereas exporters of low–value, high-mass commodities—such as ores, fertilizers, and agricultural products—will be disproportionately affected. This asymmetry may exacerbate trade inequalities, particularly for geographically isolated or developing economies reliant on primary exports. As such, global coordination mechanisms—potentially through carbon price harmonization or green fuel subsidies—may be needed to prevent the decarbonization agenda from deepening existing economic disparities.

From an implementation perspective, developing bunkering infrastructure and green corridors along major trade routes will be vital. Coordinated initiatives between governments, ports, and industry—such as the establishment of transoceanic green corridors—such as a potential China–US green corridor, which could reduce shipping emissions by 2.5%^61^—can accelerate the scale-up of alternative fuels and reduce emissions in key routes. Equally, a global alignment of standards and policies is required to prevent a patchwork of regional measures that could undermine efficiency and increase compliance costs^12^.

Overall, the study underscores that while technological pathways to decarbonize shipping are technically feasible, achieving them will demand immediate, large-scale, and coordinated action. The combination of slow fleet turnover, limited fuel infrastructure, and uncertain fuel availability means that every decade of delay narrows the window for achieving the IMO’s net-zero goals. Success will depend on coupling rapid innovation in ship technology with system-wide decarbonization of the global energy supply, ensuring that the sector’s transition unfolds in tandem with broader societal efforts to limit warming to 1.5–2 °C.

## Supplementary Information

Below is the link to the electronic supplementary material.

Supplementary Material 1

## Data Availability

The AIS data used in modelling the international shipping fleet were provided by Kystverket (Norwegian Coastal Administration) and therefore are restricted to the third party and used under license in this study. Port calls and ship data (Sea-web Ships) has been provided by IHS Markit. Weather data ECMWF Reanalysis v5 (ERA5) was provided by the Copernicus Climate Change Service (C3S) and produced and maintained by the European Centre for Medium-Range Weather Forecasts (ECMWF). Trade data used in the gravity model, including the BACI database (http://www.cepii.fr/CEPII) and UN Comtrade database (https://comtrade.un.org/data), are publicly available. The MESSAGEix model is open-source and the underlying data and code is entirely made available (https://docs.messageix.org/) 62,63 . The stock-flow model is available in its repository (https://github.com/IndEcol/ODYM). Other data and model aspects are available from the corresponding author upon request.
