# Supplementary material for: Maritime sector pathways toward net-zero emissions within global energy scenarios
Source: Sci Rep. 2026 Feb 11;16:8282. doi: 10.1038/s41598-026-35909-4 (PMC12966394; doi:10.1038/s41598-026-35909-4)
Supplement: Supplementary file 1 — Supplementary Material 1 [file 41598_2026_35909_MOESM1_ESM.docx]

Supplementary Information: Maritime sector pathways towards net-zero emissions within global energy scenarios

Diogo Kramel*^1^, Volker Krey^1,2^, Oliver Fricko^2^, Florian Maczek^2^, Helene Muri^1^, and Anders H. Strømman^1^

^1^Industrial Ecology Programme (IndEcol), Norwegian University of Science and Technology (NTNU), Trondheim, Norway

^2^International Institute for Applied Systems Analysis (IIASA), Vienna, Austria

* Corresponding author: diogo.kramel@ntnu.no

Contents

[Supplementary Methods 1](#_Toc213420947)

[Supplementary Note 1 6](#_Toc213420948)

[Supplementary Note 2 7](#_Toc213420949)

[Supplementary Note 3 9](#_Toc213420950)

[Supplementary Note 4 10](#_Toc213420951)

[Supplementary Note 5 11](#_Toc213420952)

[Supplementary References 11](#_Toc213420953)

# Supplementary Methods


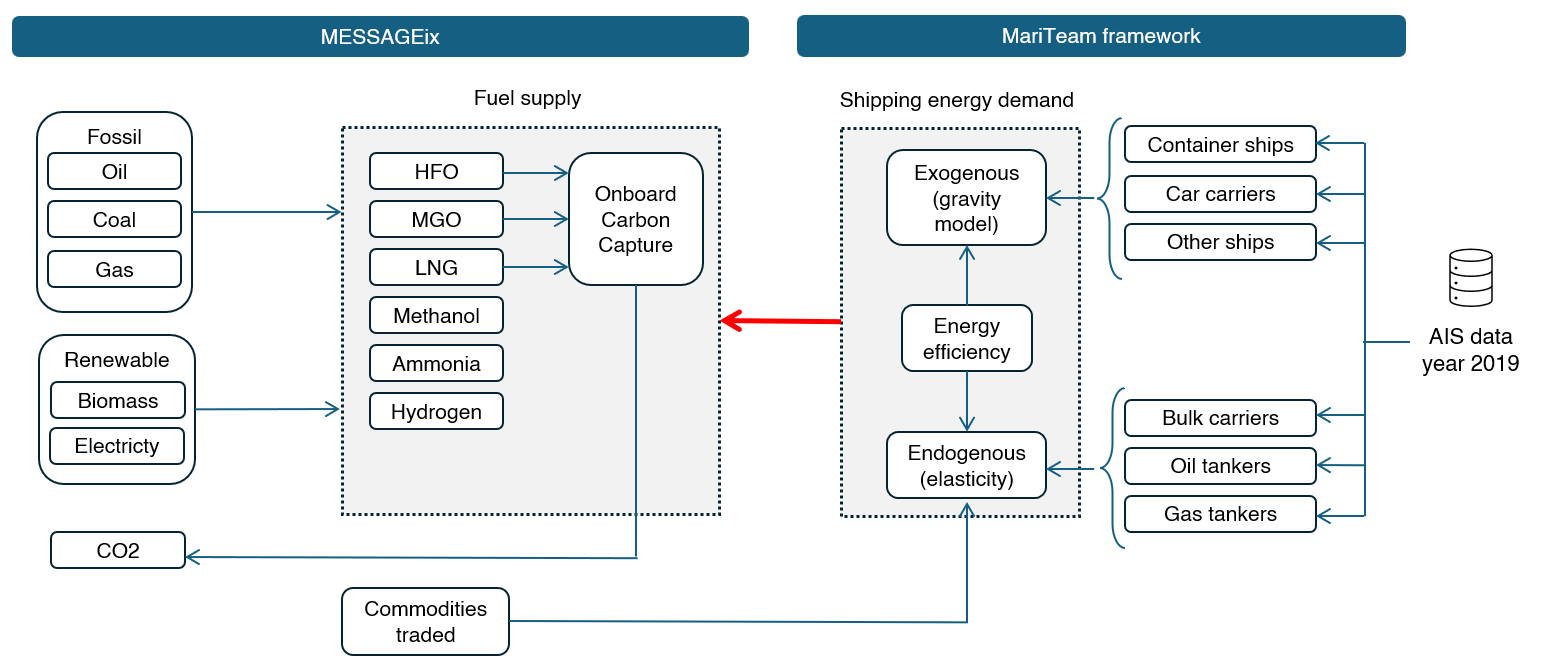


**Figure 1**: Flowchart illustrating the linkage between MariTeam and MESSAGEix implemented as a soft, unidirectional coupling: energy demand trajectories estimated by MariTeam serve as inputs to MESSAGEix.

The MariTeam model (Maritime Transport Environmental Assessment Model) is a bottom-up framework for ship emission assessment that integrates ship positional data with technical vessel specifications and ship resistance models to estimate the energy demand and associated emissions from the maritime sector (Kramel et al. 2021; Kim et al. 2023).

The model employs Automatic Identification System (AIS) data to determine ship positions (latitude and longitude) and speed over time, as recorded by terrestrial receivers and the NorSat-1 and NorSat-2 satellites. The dataset comprises approximately 15 billion AIS messages collected between 2018 and 2024, representing 88,000 vessels. To account for weathers conditions, each AIS observation is linked with wind and sea state parameters from the ERA-Interim reanalysis produced by the European Centre for Medium-Range Weather Forecasts (ECMWF). Technical ship characteristics, including hull and engine dimensions required for ship resistance modelling, are obtained from the Sea-web Ships database, while missing parameters are estimated following the methodology of Kim et al. (2022). Ship resistance is subsequently evaluated using a meta-model developed by Kim et al. (2023), which integrates calm-water resistance with additional wind and wave resistance components. Together, these elements provide the foundation for constructing a global inventory of shipping energy demand and emissions, as seen in Figure 1.

*
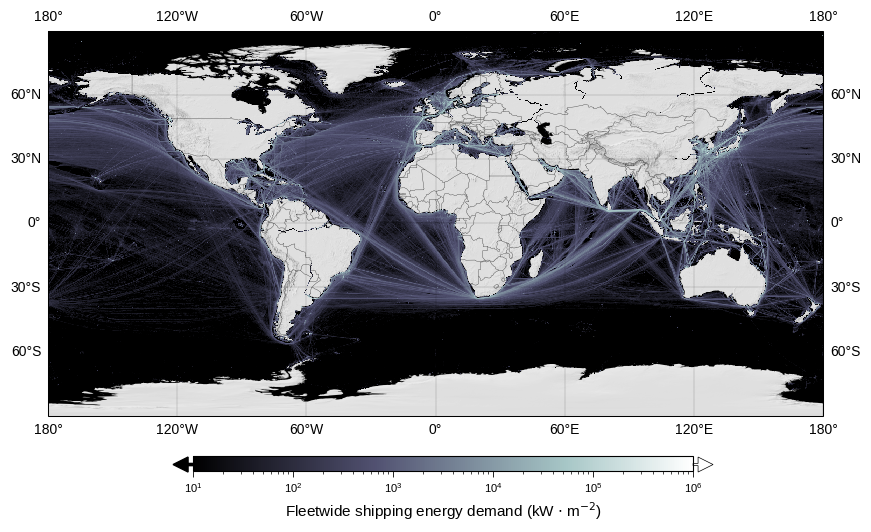
*

**Figure 2**: Geographical distribution of annual average energy consumption (kW km^-2^) from global shipping from the MariTeam model for 88,000 ships including merchant and working vessels engaged in domestic and international voyages averaged for years 2018 to 2021. Data source: authors’ own from Kramel et al. (2021)^1^.

First, we compare the demand per ship type with the 4^th^ IMO GHG Study^2^. Figure 2 depicts global fleet fuel demand across vessel categories, for the MariTeam model in 2019 with the findings from the 4th IMO GHG^2^ study for the year 2018. The colored bars represent the MariTeam model's estimates, while the corresponding dots overlaying each bar display the IMO's study results. This juxtaposition highlights the agreement with minor variations in the two sources, indicative of methodological differences or temporal changes in fleet composition and energy efficiency between 2018 and 2019.


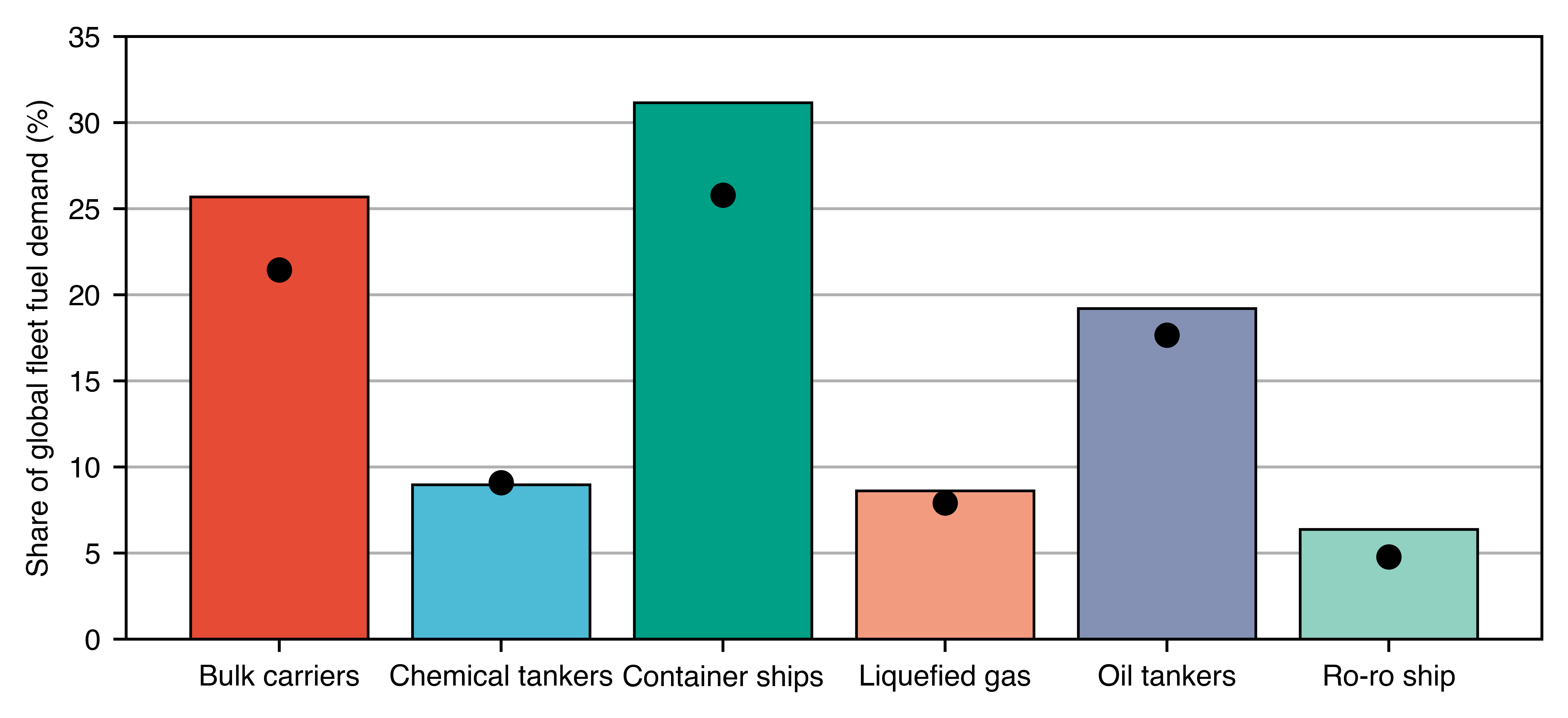


**Figure 3**: Comparison of shipping energy demand between the MariTeam model (bars)
in 2019 and the 4th IMO GHG study^2^ (dots) in 2018.

In order to capture the limitation of liquified hydrogen penetration in the global shipping fuel mix, we divide the total energy demand based on what we consider an attainable maximum distance voyage that could be achieve by using exclusively hydrogen internal combustion engine (H2ICE). Thus, total energy demand is disaggregated in short-haul, distances shorter than 1,000 km and long-haul, distances longer than 1,000 km. Figure 3 display the distribution of energy demand among different ship types for short- and long-haul voyages in 2019. This chart underscores the significant variance in energy requirements across ship types, reflecting operational, structural, and voyage distance differences.


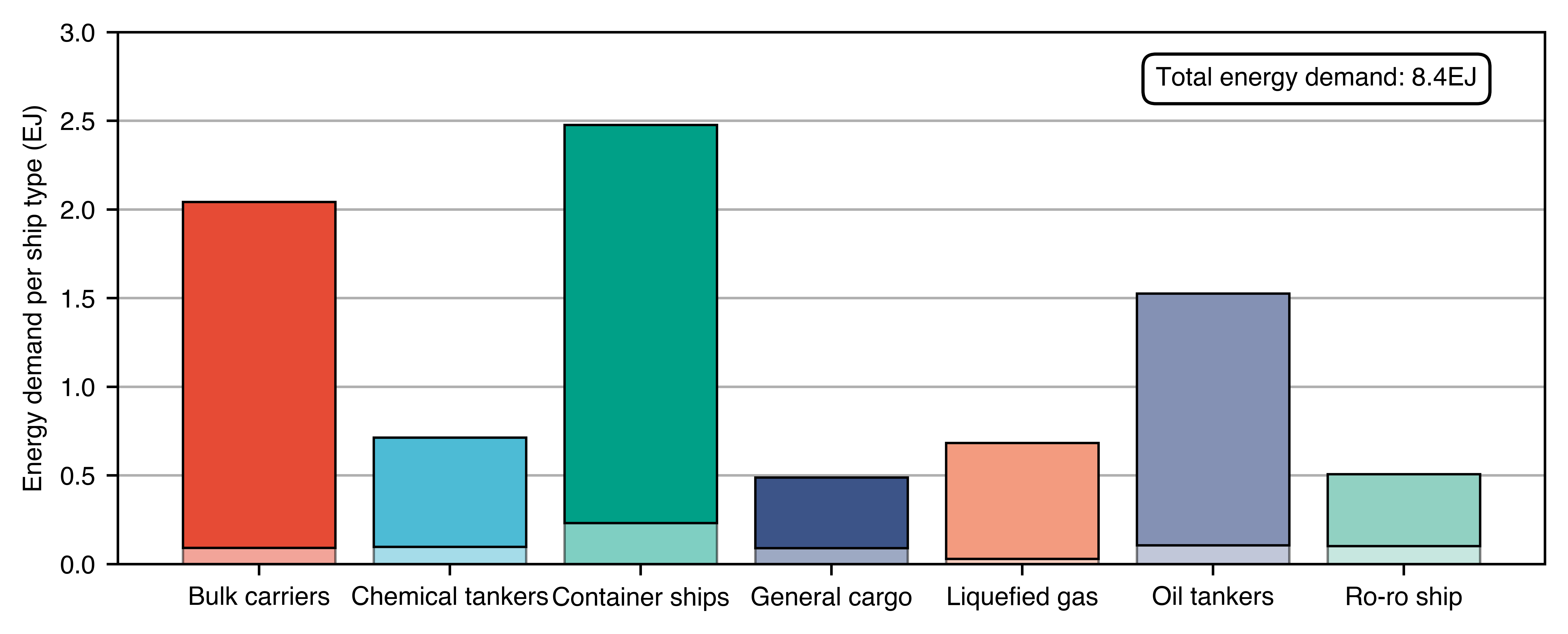

**Figure 4**: Shipping energy demand for short- (bottom, in lighter colours) and long-haul (top) voyages in 2019
based on the MariTeam model.

To build shipping energy demand projections, we use scenarios developed by Kramel et al. (2024)^3^ that makes use of gravity models to create shipping energy demands aligned with the SSP-RCP framework. Figure 4 illustrates the relative growth in energy demand for various ship types over an 80-year horizon, as modeled in the SSP2 scenario, for the aforementioned publication. Energy demand is based on year 2020 using pre-COVID data from 2019. Trajectories in Figure 4 are indexed to the base year of 2020, with subsequent years depicted as a percentage increase from this reference point. The general trend across all ship types is a consistent increase in energy demand, underscoring the maritime sector's expanding energy needs in the context of global economic development and trade patterns anticipated in the SSP2 scenario. The dashed lines in ship types (i.e., chemical tanker, liquefied gas, and oil tankers) represent the ship segments that will be adjusted by endogenous demands of coal, oil, gas and high-value chemicals (HVC) in MESSAGEix.


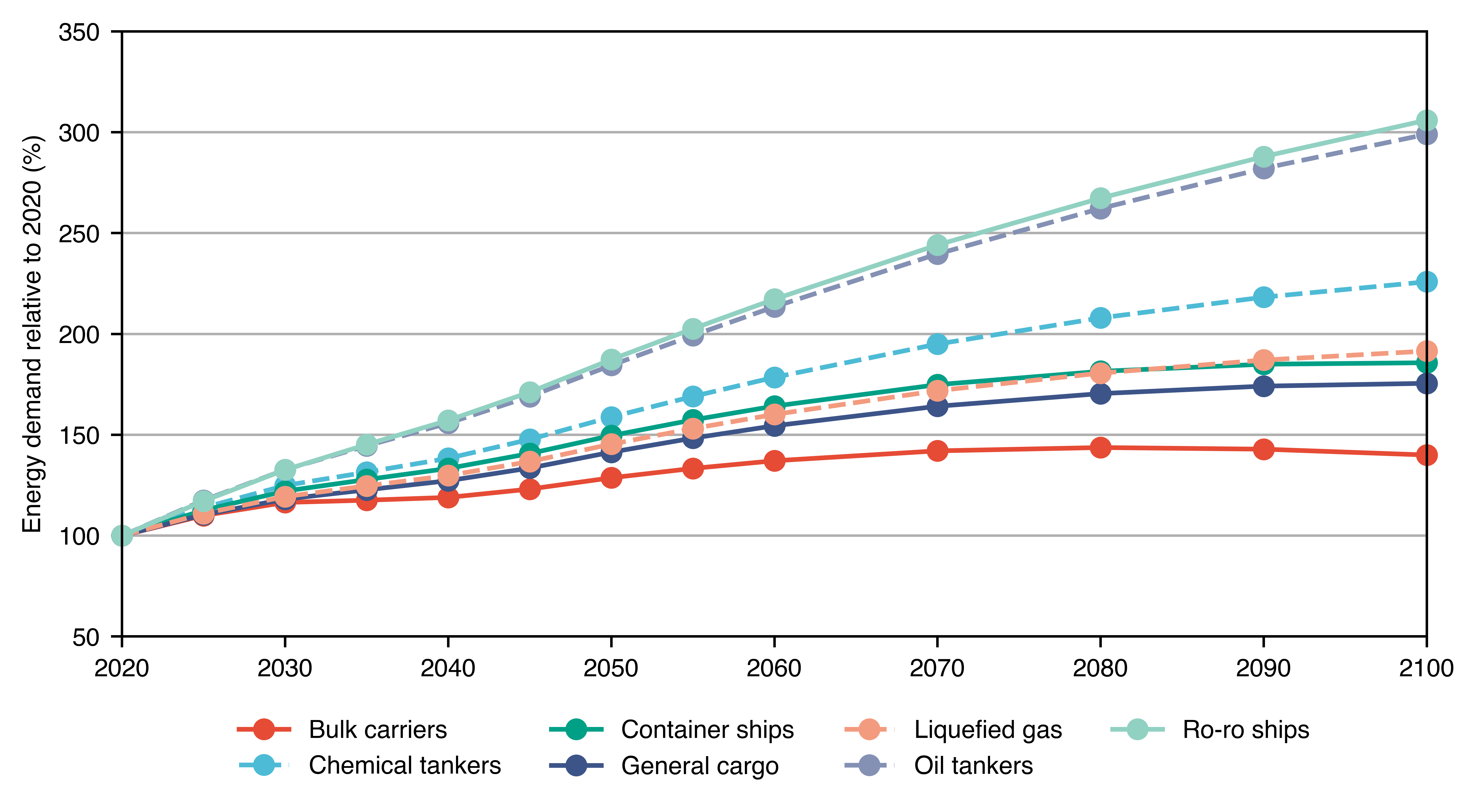

**Figure 5**: Growth trajectories for different ship types in the SSP2 scenario
based on the gravity model of trade for the 2020-2100 time range.

The energy demand in the baseline year 2019, show in Figure 2, is then combined with gravity model projections show in Figure 4. Figure 5 displays the projected energy demand trajectory for different types of ships within the SSP2 scenario, spanning from 2020 to 2100. This projection indicates a continuous increase in the energy demand for the shipping sector throughout the century.


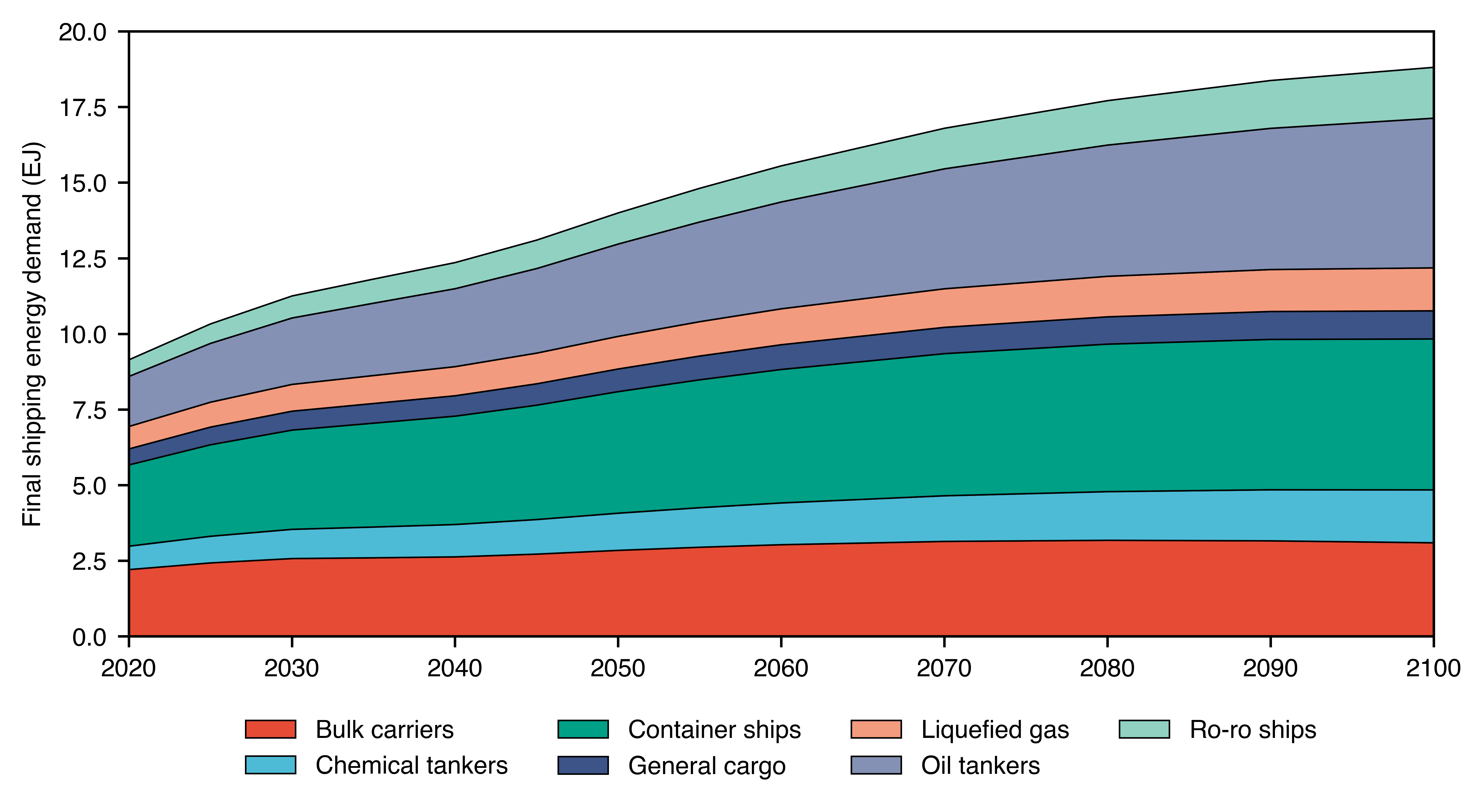

**Figure 6**: Result shipping energy demand per ship type for the SSP2 scenario
for the 2020-2100 time range.

The energy demand shown will be used demand parameter for international shipping in MESSAGEix in a 5 year resolution between 2020 and 2060, and 10 years resolution between 2060 and 2110.

We apply a dynamic stock model to investigate at what pace shipping can transition from the current fuel mix to alternative fuels. Figure 6 portrays the historical trends in shipbuilding over approximately four and a half decades, beginning in 1970 and concluding around 2015. The ship data is obtained from the IHS Sea-Web database. We use for that the ship type and the built year to estimate the number of new build ships annually.


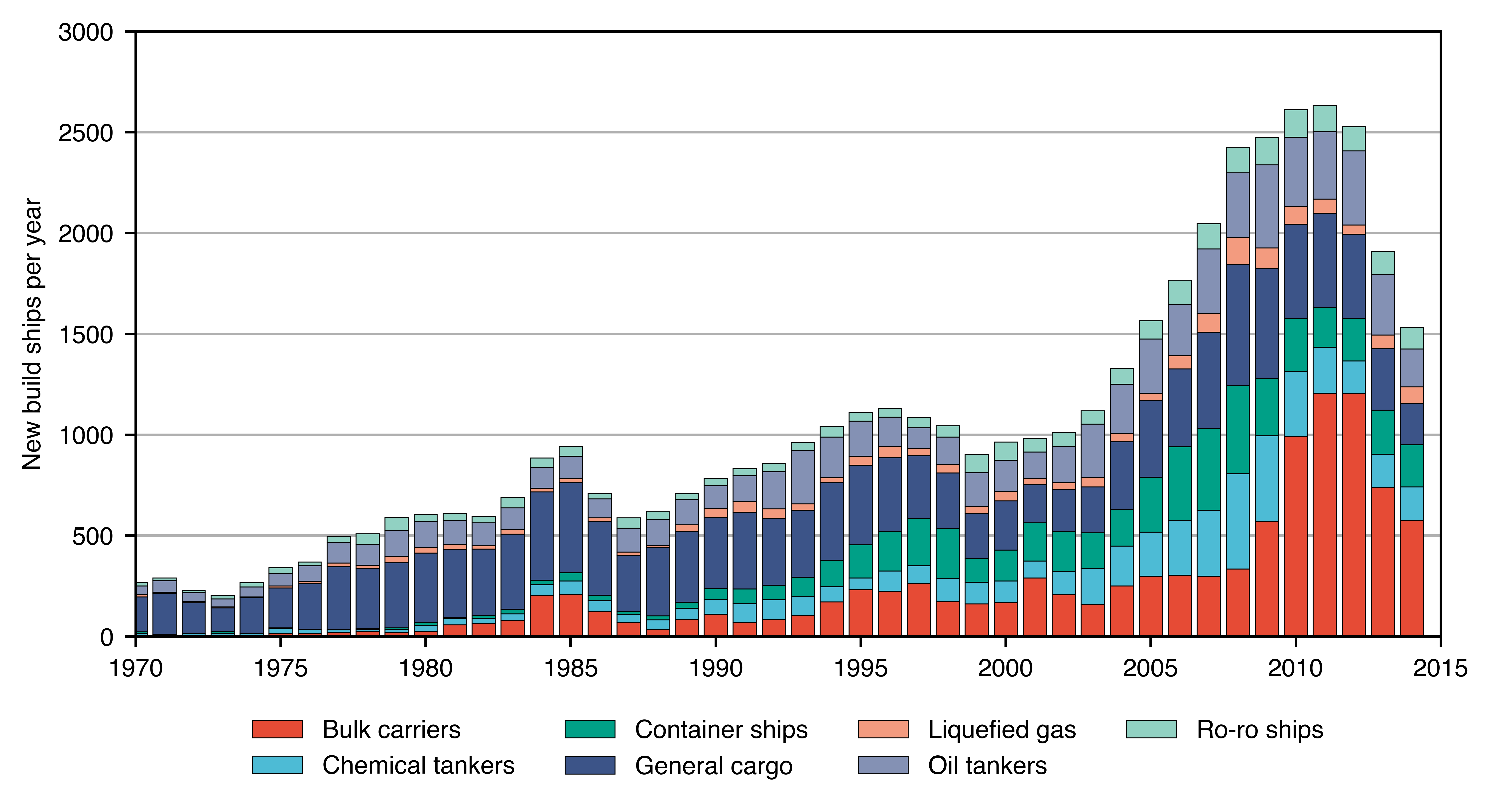

**Figure 7**: Historic ship data for new build ships from 1970 to 2015.

We use a dynamic stock-flow model^4^ to reconstruct historical and future inflows and outflows in the shipping fleet, as show in Figure 7. We model the retirement of the ships as a normal distribution of 25 years and standard deviation of 5 years^3^.


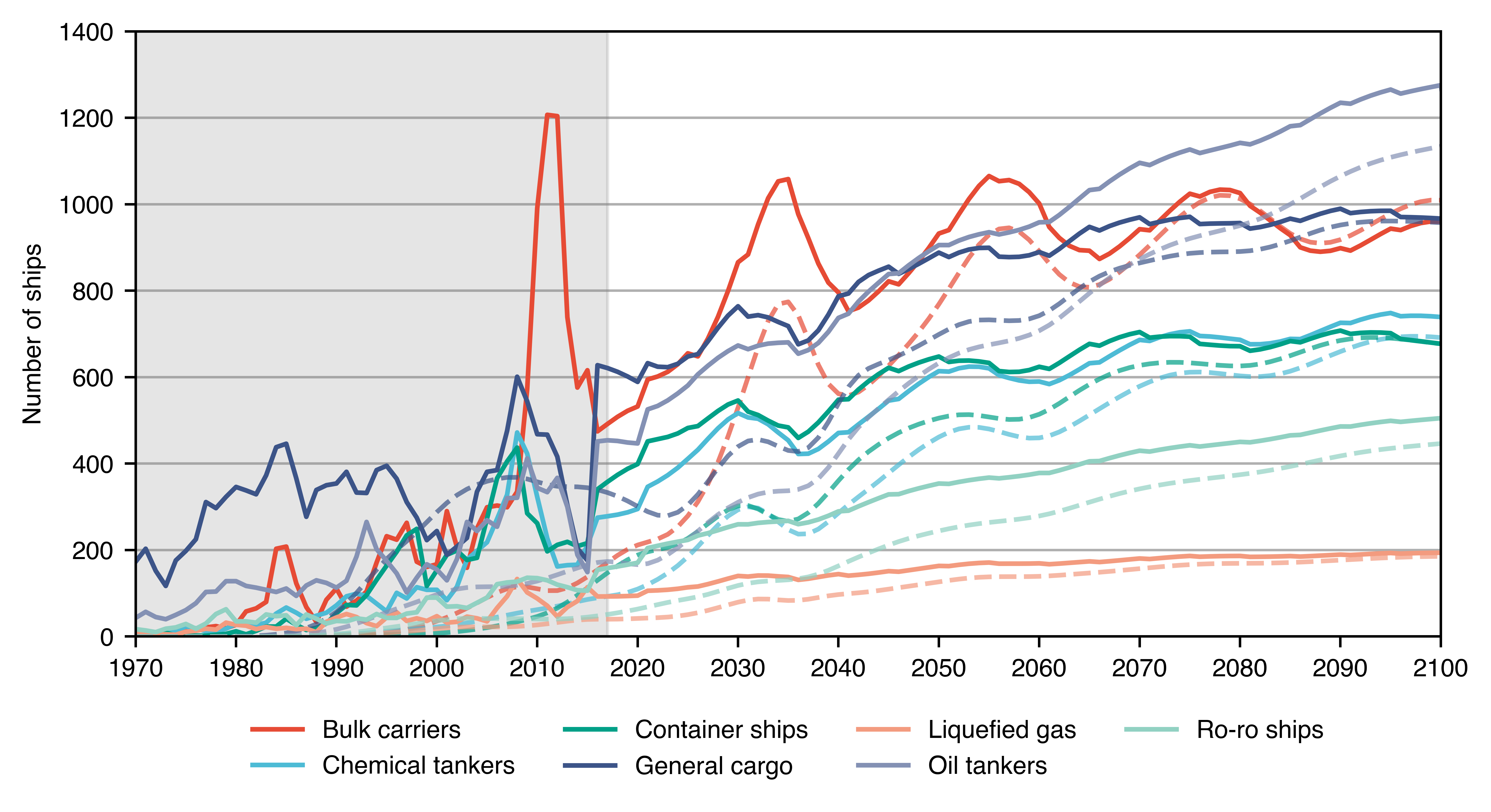

**Figure 8**: Inflows (continuous) and outflows (dashed) of ships based on historic data and
stock model from 1970 to 2100. Shaded area represents historic period.

The phase-out process of the current fleet together with the phase-in of new ships is show in Figure 8. Because of a peak in the orderbook or bulk carriers, general cargo ships, chemical tanker and container ships between 2005 and 2015, one can see fluctuations in the flows in the subsequent decades.


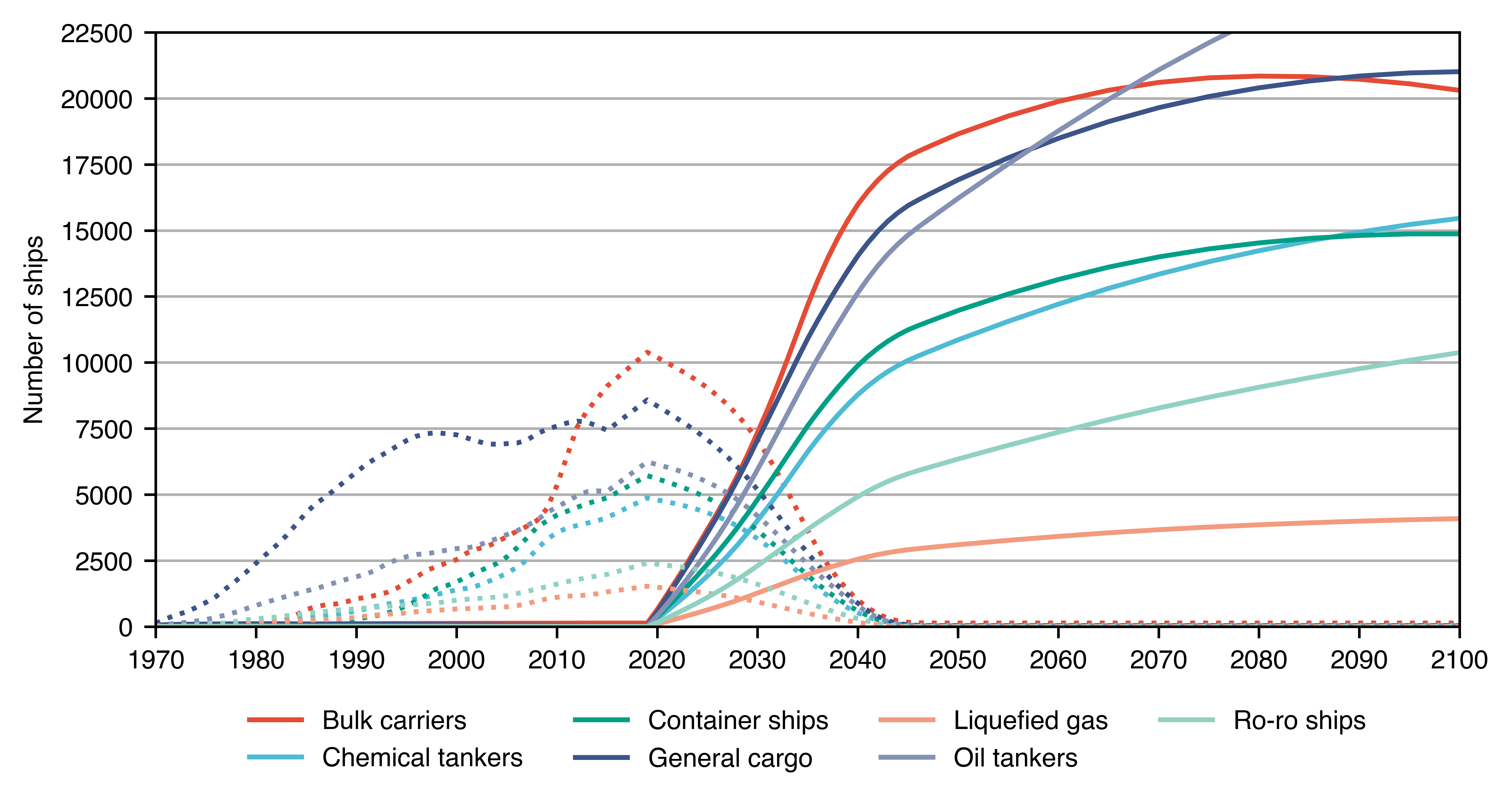

**Figure 9**: Ship stock change from current fleet (dashed) to
new built vessels (continuous) from 1970 to 2100.

# Supplementary Note 1

Gains in energy efficiency across the fleet can be a major strategy in reducing emissions in the sector by decreasing the demand for fuels. To estimate the potential gains, we use the lower quartile of the CO_2_ emission reduction potential in Bouman et al. (2017)^5^ for hull design, power & propulsion, and operational measures. The efficiencies applied are shown in Table 1.

**Table 1**: Energy reduction potential in terms of hull design and power & propulsion
according to median values of Bouman et al. (2017).

|  | **Measure** | **Potential efficiency gain** |
| --- | --- | --- |
| **Hull design** | Hull shape | 5% |
|  | Air lubrication | 3% |
|  | Hull coating | 1% |
| **Power and propulsion** | Power system | 3% |
|  | Propulsion system | 2% |
|  | Onboard generation | 1% |
| **Operational measures** | Voyage optimization | 5% |
|  | Speed optimization | 8% |

If all strategies above are deployed simultaneously, their combined cumulative efficiency gains could reach 25%. Because they would be unfeasible to be applied in existing ships or would demand extensive retrofitting, the energy efficiency gains are applied only to the newly built vessels. Results are shown in Figure 9.

**
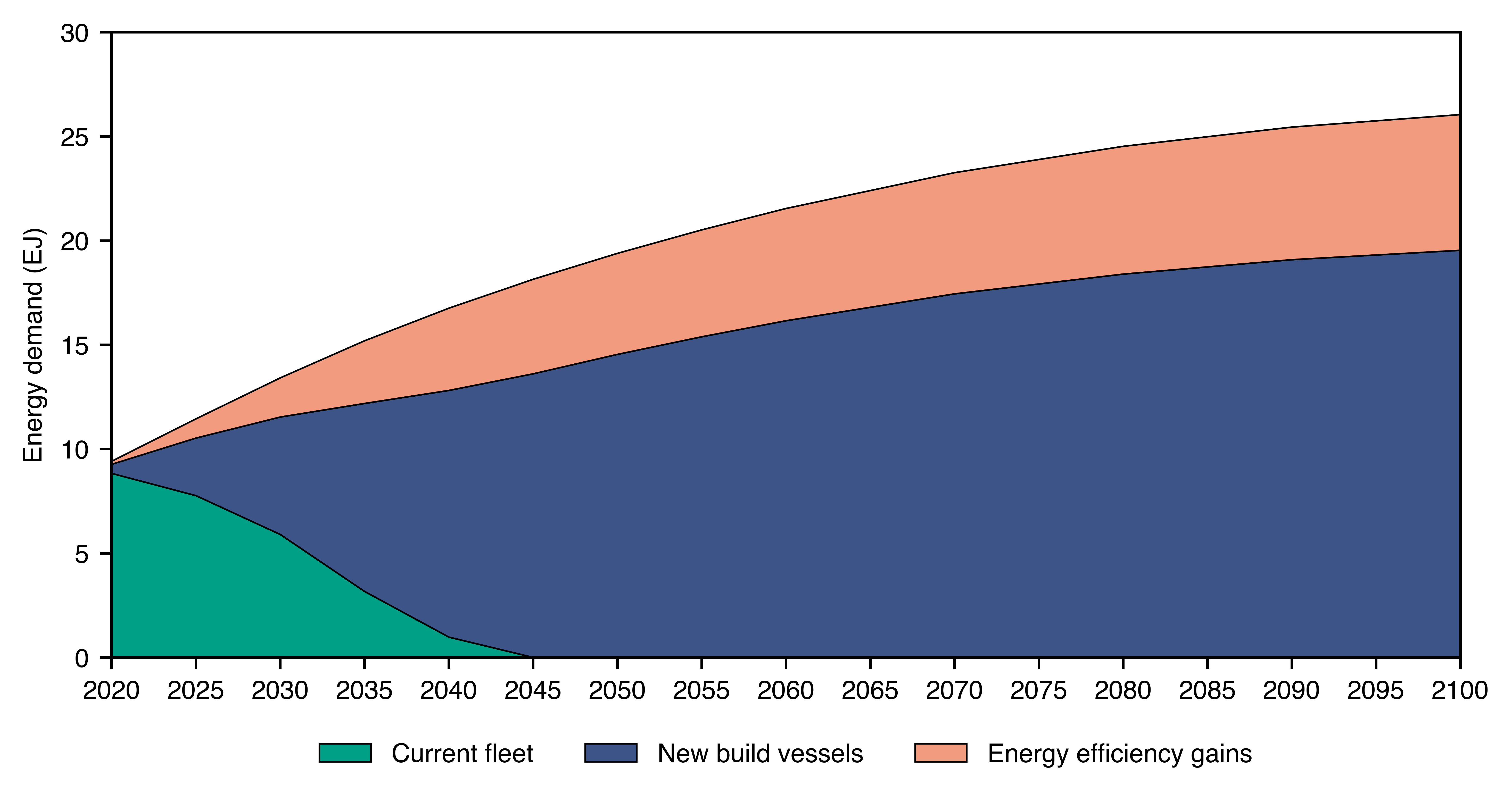
**

**Figure 10**: Energy demand for ship cohort and gains with new-built vessels' energy efficiency.

# Supplementary Note 2

The emissions are categorized by pollutant type and include carbon dioxide (CO_2_), methane (CH_4_), nitrous oxide (N_2_O), volatile organic compounds (VOCs), nitrogen oxides (NOx), carbon monoxide (CO), black carbon (BC), ammonia (NH3), sulfur dioxide (SO_2_), and organic carbon (OC). The fuels assessed are Heavy Fuel Oil (HFO), Marine Gas Oil (MGO), Liquefied Natural Gas (LNG), Methanol (Meth), Ethanol (Eth), Ammonia (NH_3_), and Liquid Hydrogen (LH_2_). Figure 9 presents a comparative analysis of the upstream emissions profile in grams per kilowatt-hour (g/kWh), allowing for a direct comparison of the environmental impact of each fuel type in terms of specific pollutants.


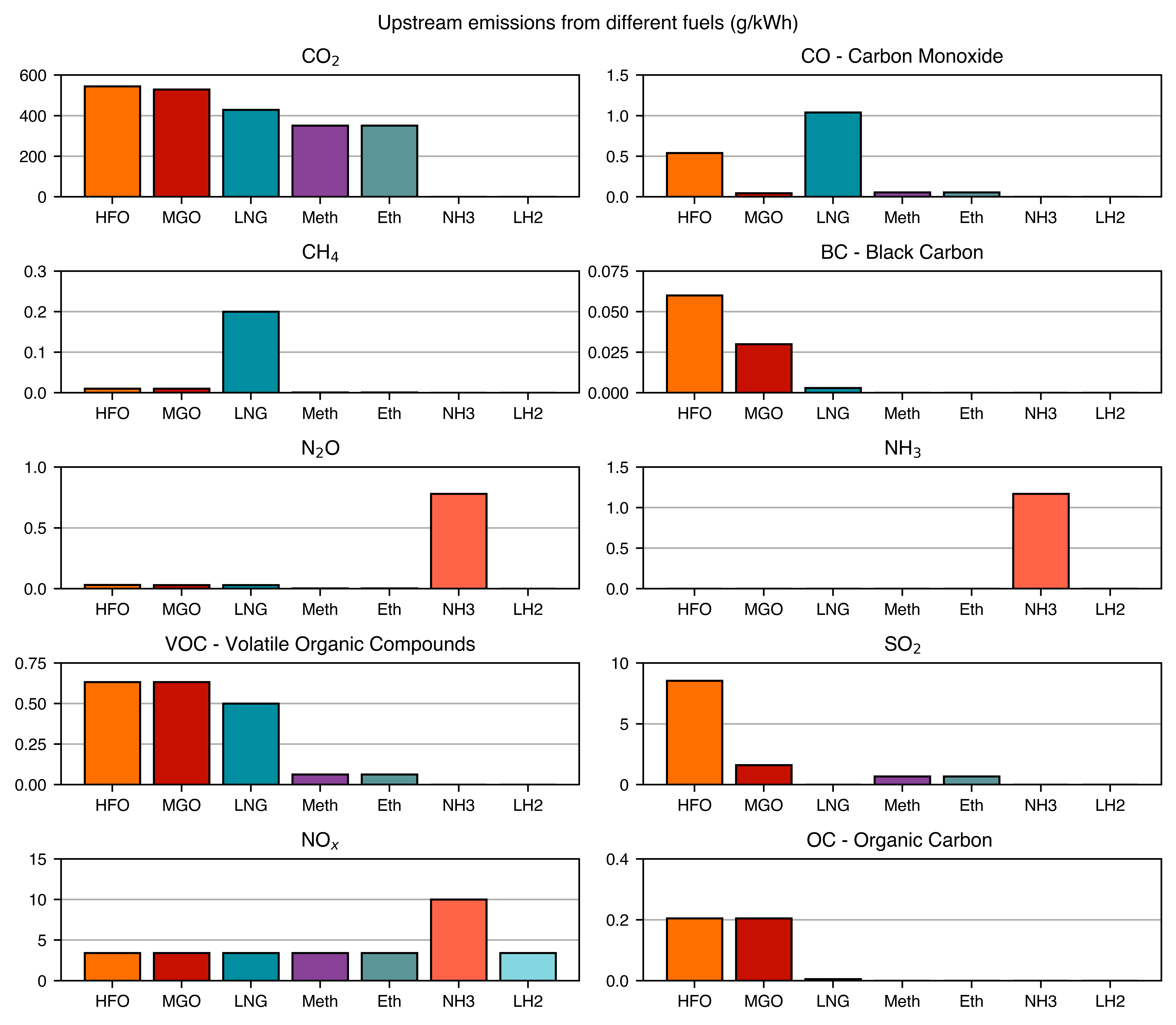


**Figure 11**: Direct ship emissions for GHG and aerosols across different fuels.

Figure 10 illustrates the projected trajectory for the reduction in emission factors of ammonia-fueled engines over the course of the 21st century. Emissions are based on the work of Schwarzkopf et al. (2023)^6^. The emission factor, expressed in grams per kilowatt-hour (g/kWh), starts at approximately 0.778 g/kWh in 2020 and falls to 0.015 g/kWh in 2015. The values are based on Schwarzkopf et al. (2023)^6^ assumptions to a case implementing an uncontrolled ammonia engine technology (compression ignition engine with marine gas oil (MGO) as pilot fuel) versus controlled technology (a spark ignition engine using hydrogen as the pilot fuel and exhaust gas treatment). It then demonstrates a steady decline, ultimately approaching zero emissions slightly beyond the 2080 mark. This trend line is indicative of the anticipated advancements in engine technology and emission control strategies, which are projected to significantly mitigate the environmental impact of ammonia fueled engines.


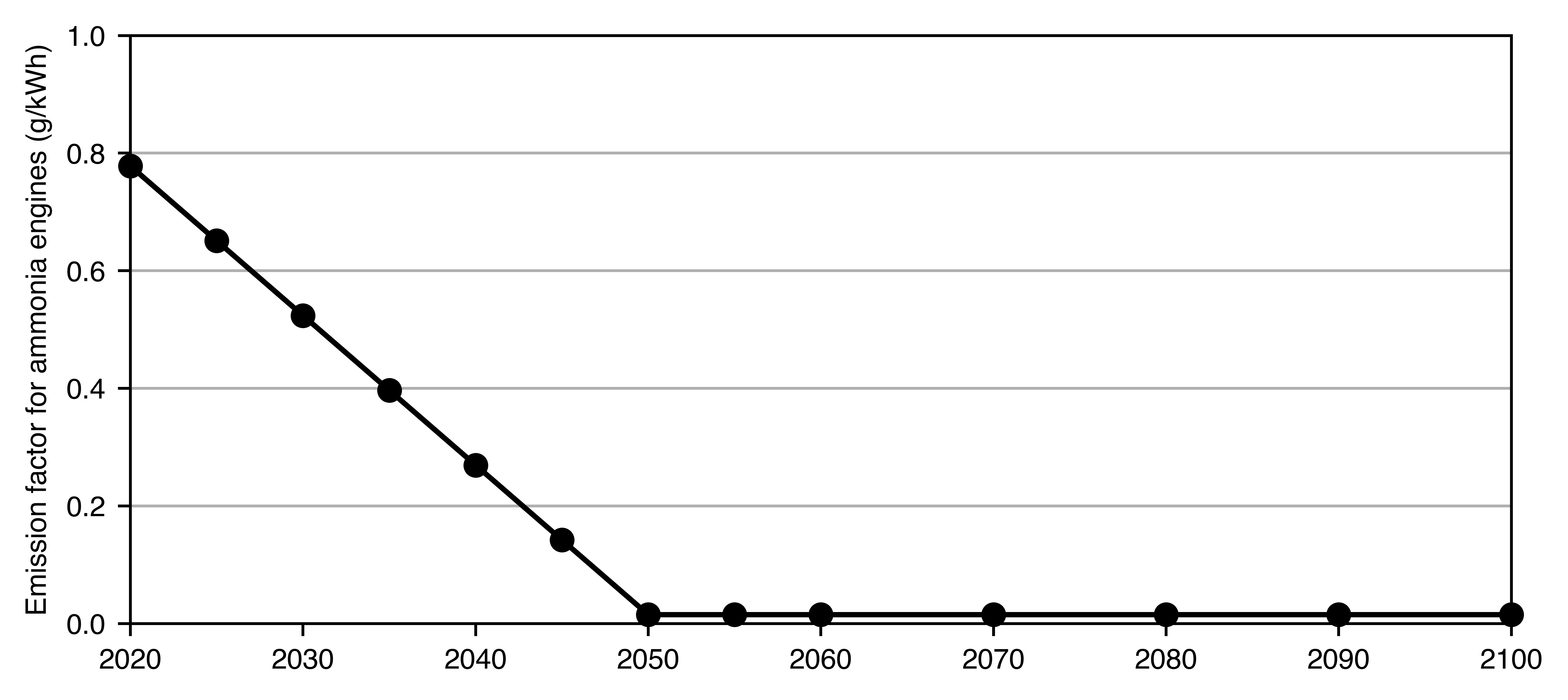


**Figure 12**: Prospective emissions for N2O in ammonia engines starting at a pessimistic case of diesel as pilot fuel and moving to hydrogen as pilot fuel by 2050.

# Supplementary Note 3

LULUC (Land use and land-use change) emissions for biomass cultivation and biofuel production are derived from two experiments carried out with the MESSAGEix-GLOBIOM framework, so we can ensure emission factors are internally consistent with other fuel pathways. We use the same procedure as adopted by the Sixth Assessment Report (AR6)^7^ of the IPCC to derive emissions associated with primary biomass supply using stylized scenarios from the EMF-33^8^.

For that, LULUC emissions are taken by the difference of a baseline scenario and a scenario running with no bioenergy demand. The baseline scenario is a scenario limiting peak warming to “likely below 2^o^C”. Biomass demand in this scenario is approximately twice as the “no bioenergy” scenario by the end of the century (200EJ versus 90EJ). As food production demand remains the same in both scenarios, we can assume the difference in biomass demand is destined to biofuel production and the difference in AFOLU (Agriculture, Forestry and Other Land Use) emissions shall be representative of the total emissions for biofuel production. Thus, we divide the cumulative AFOLU emissions between 2020 and 2100 by the cumulative bio-energy production over the 2020-2100 period, from which we obtain 19gCO2eqMJ^-1^.

a)**
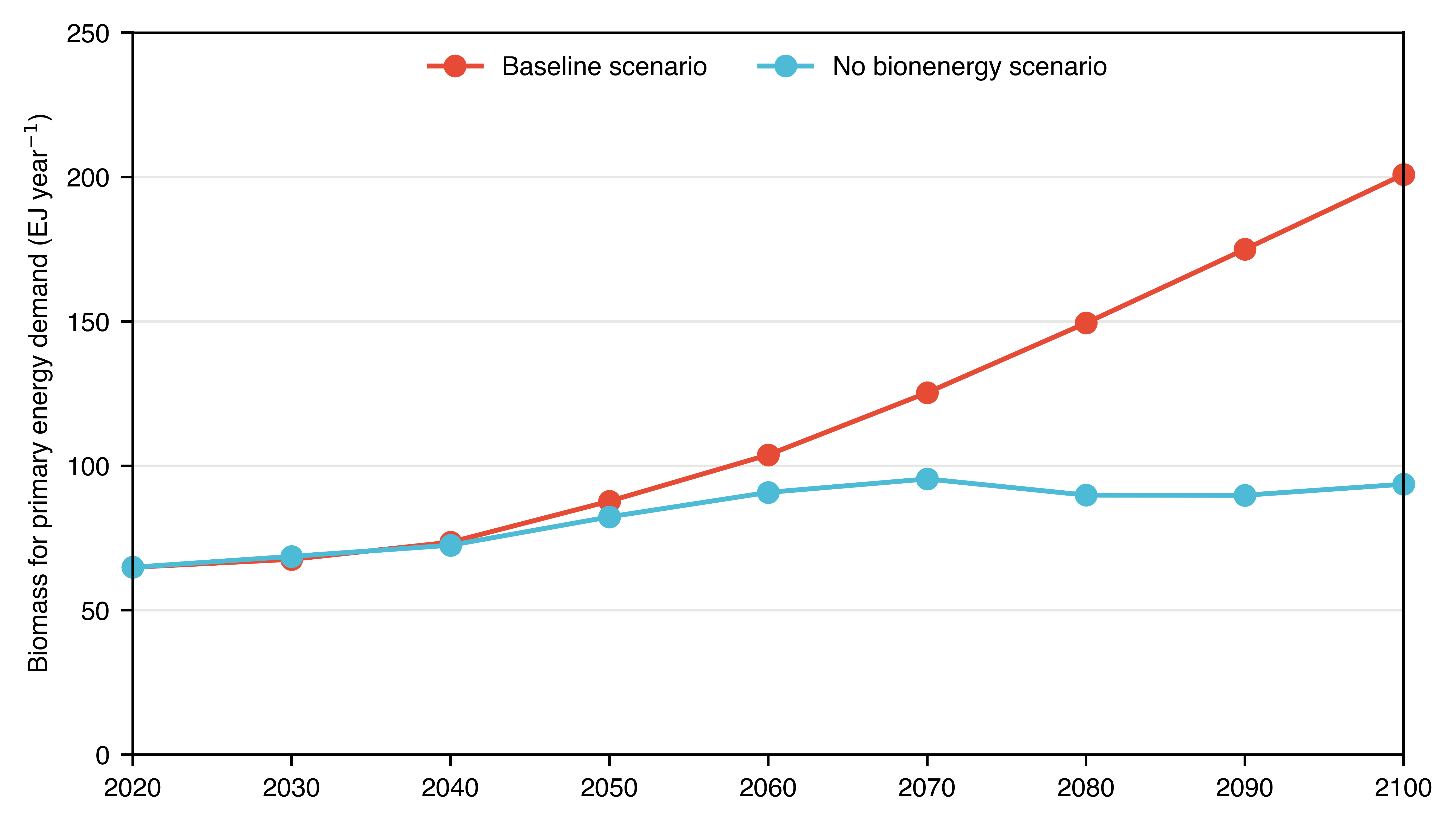
**

b)
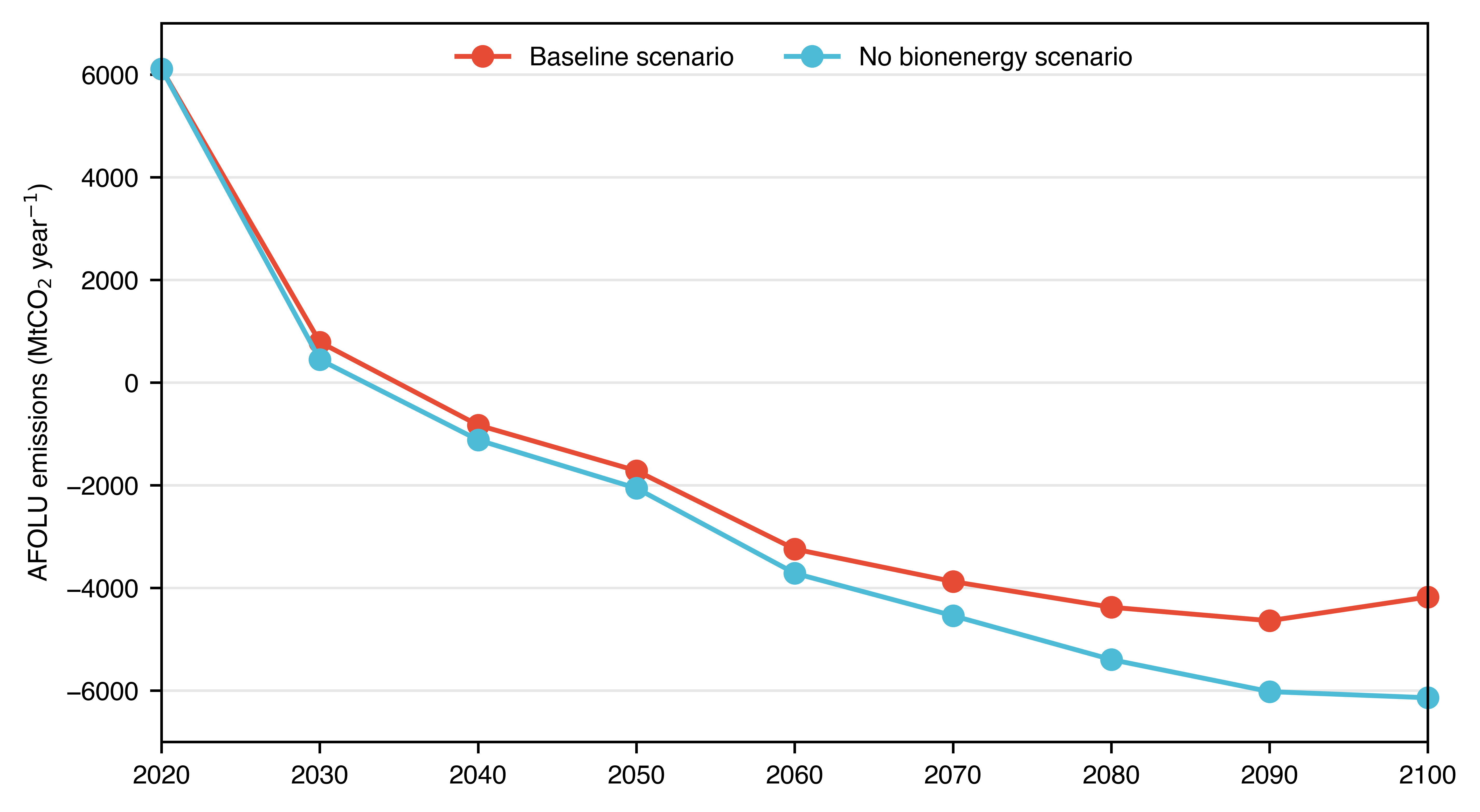


**Figure 13**: Biomass for primary energy and AFOLU emissions for a baseline scenario (C4 scenario or “likely below 2^o^C”) and a scenario variant but with no bioenergy made available. Figure (a) displays the biomass for primary energy demand and (b) shows the resulting AFOLU emissions.

# Supplementary Note 4

The adoption of biofuels as an alternative fuel presents significant environmental and social implications. Thus, we explore the implications for food security and biodiversity conservation by comparing three scenarios: business as usual (no climate targets in place) and the B600 and B1000 scenarios described in the main manuscript.

The competition for land between biofuel crops and food crops can threaten food security, particularly in developing nations. Our comparison shows that would have implications in terms of population under the risk of hunger (see Figure 13a). However, the expansion of biocrops plantations can lead to habitat loss and degradation, posing risks to biodiversity. Our scenario comparison shows that by the end of the century total land cover for natural forests could be reduced from 2.75 billion hectares to 2.1 and 1.9 billion hectares in the B600 and B1000 scenarios.

a)
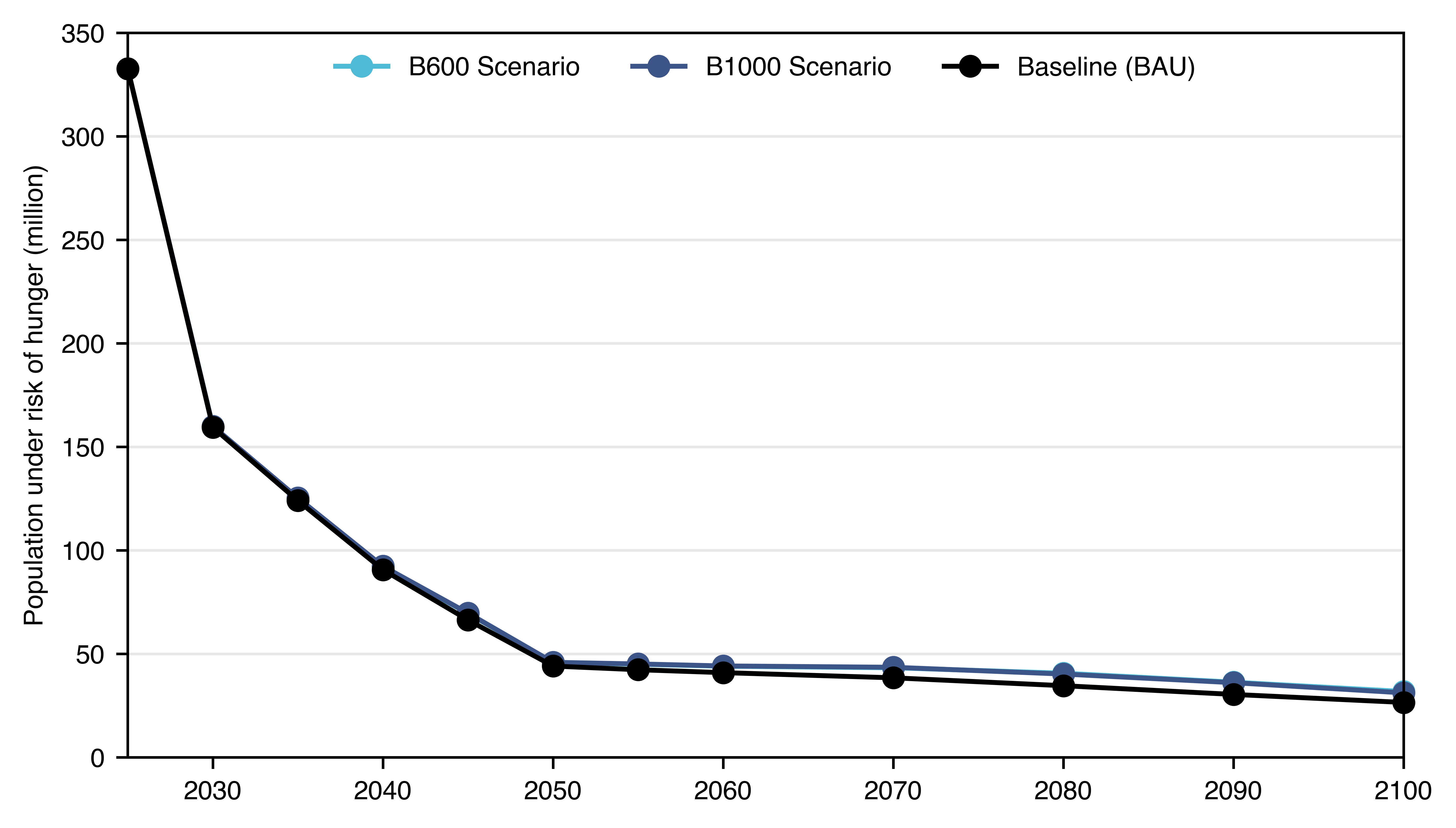


b)**
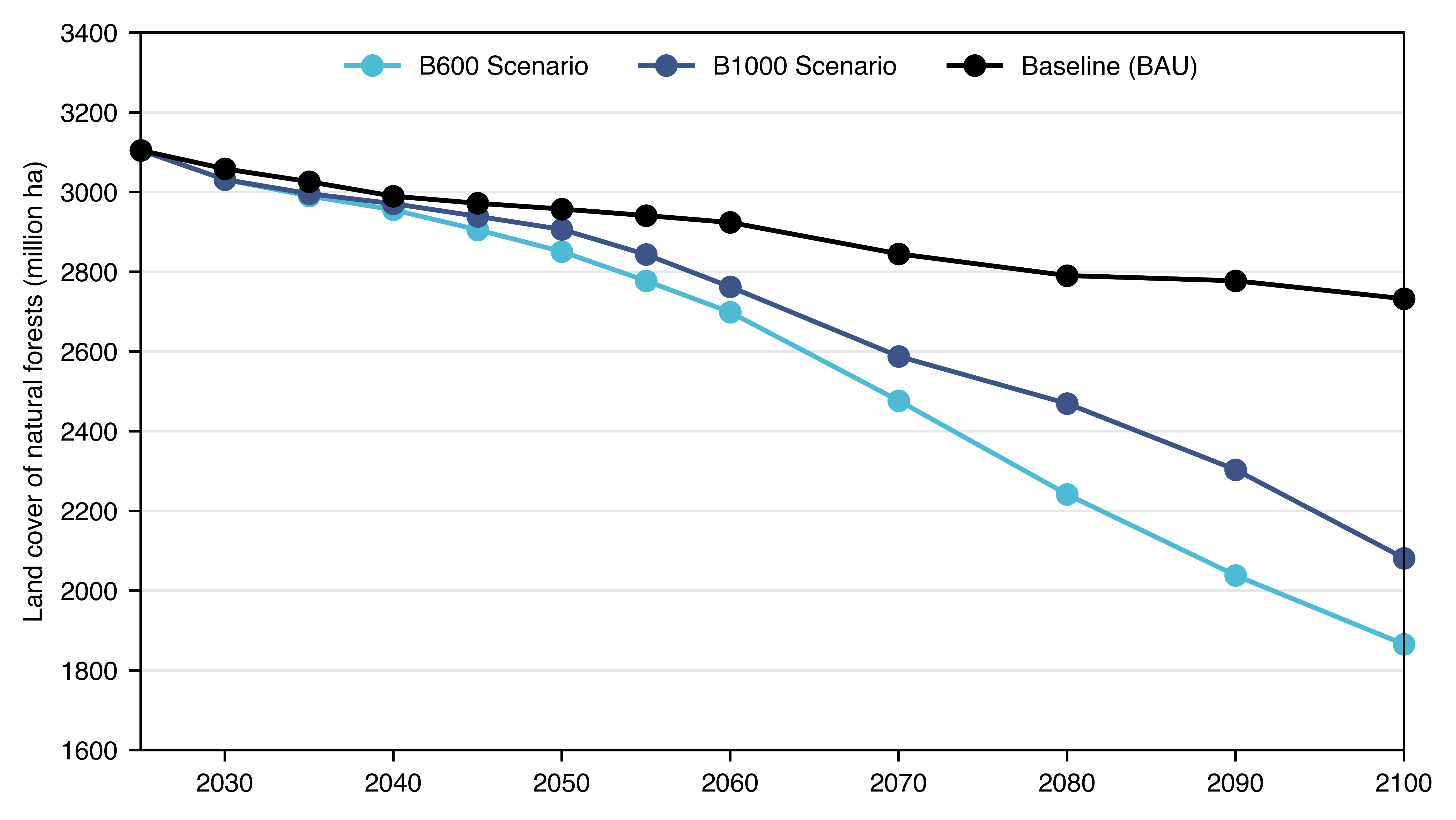
**

**Figure 14:** Scenario comparison between and business-as-usual scenario (not climate policies) and the B600 and B1000 scenarios used in this study for food security in (a) and biodiversity conservation in (b).

# Supplementary Note 5

Summary of techno-economic parameters assumption for different fuel technologies.

| Fuel | Parameter | Value | Reference |
| --- | --- | --- | --- |
| HFO | Fuel penalty when operating with OCC | 12% | ^9^ |
|  | Carbon capture rate of OCC | 75% | ^9^ |
|  | Cost of OCC per tonne of fuel | US$36 | ^10^ |
| MGO | Fuel penalty when operating with OCC | 12% | ^9^ |
|  | Carbon capture rate of OCC | 75% | ^9^ |
|  | Cost of OCC per tonne of fuel | US$36 | ^10^ |
| LNG | Fuel penalty when operating with OCC | 9% | ^9^ |
|  | Carbon capture rate of OCC | 85% | ^9^ |
|  | Cost of OCC per tonne of fuel | US$40 | ^10^ |
| LH2 | Maximum sailing range | 1000km | Experts |
| NH3 | Deployment year of ammonia engines | 2030 | Experts |
| Biofuels | LULUC emissions | 19gCO2eqMJ-1 | Own calculations |
| NA | Average ship lifetime | 25 years | Experts |
|  | Overall ship energy efficiency improvement | 25% | Own calculations |

# Supplementary Note 6

Increase in trading costs by region.

| Category | Raw materials | Agriculture | Manufacturing | Crude oil | **Average** |
| --- | --- | --- | --- | --- | --- |
| Global north | 14.6% | 8.1% | 5.6% | 10.7% | **9.8 %** |
| Global south | 17.8% | 9.8% | 8.4% | 11.4% | **11.9 %** |
| North America | 13.5% | 8.6% | 5.3% | 11.3% | **9.7 %** |
| South America | 19.5% | 9.6% | 7.0% | 10.6% | **11.7 %** |
| South-east Asia | 16.2% | 9.9% | 8.9% | 13.0% | **12.0 %** |
| Africa | 18.4% | 9.7% | 9.0% | 10.3% | **11.9 %** |
| Europe | 10.4% | 5.6% | 3.7% | 10.7% | **7.6 %** |

# Supplementary Note 7

A list of key acronyms used in the publication.

| IMO | International Maritime Organization |
| --- | --- |
| MEPC | Marine Environment Protection Committee |
| LCA | Life-cycle Assessment |
| IAM | Integrated Assessment Model |
| SSP | Shared Socioeconomic Pathways |
| RCP | Representative Concentration Pathways |
| GHG | Greenhouse Gas |
| IPCC | Intergovernmental Panel on Climate Change |
| OCCS | Onboard Carbon Capture and Storage |
| BECCS | Bioenergy with Carbon Capture and Storage |
| HFO | Heavy Fuel Oil |
| MGO | Marine Gasoil |
| LNG | Liquefied Natural Gas |
| LH2 | Liquefied Hydrogen |
| NH3 | Ammonia |
| TRL | Technology Readiness Level |
| LULUC | Land-use and Land-use-change |
| WTT | Well to Tank |
| TTW | Tank to Wake |
| WTW | Well to Wake |

#

# Supplementary References

1. Kramel, D. *et al.* Global Shipping Emissions from a Well-to-Wake Perspective: The MariTEAM Model. *Environ. Sci. Technol.* **55**, 15040–15050 (2021).

2. Faber, J., Shinichi, Hanayama, S., Zhang & Paula, Pereda, B., Comer. Fourth IMO GHG Study. (2020).

3. Kramel, D. *et al.* Advancing SSP-aligned scenarios of shipping toward 2050. *Sci. Rep.* **14**, 8965 (2024).

4. Pauliuk, S. & Heeren, N. ODYM—An open software framework for studying dynamic material systems: Principles, implementation, and data structures. *J. Ind. Ecol.* **24**, 446–458 (2020).

5. Bouman, E. A., Lindstad, E., Rialland, A. I. & Strømman, A. H. State-of-the-art technologies, measures, and potential for reducing GHG emissions from shipping – A review. *Transp. Res. Part Transp. Environ.* **52**, 408–421 (2017).

6. Schwarzkopf, D. A. *et al.* Future Ship Emission Scenarios with a Focus on Ammonia Fuel. *Atmosphere* **14**, 879 (2023).

7. Nabuurs, G.-J. *et al.* Agriculture, forestry and other land uses (AFOLU). in *Climate Change 2022: Mitigation of Climate Change.* 747–860 (Cambridge University Press, 2023).

8. Rose, S. K. *et al.* An overview of the Energy Modeling Forum 33rd study: assessing large-scale global bioenergy deployment for managing climate change. *Clim. Change* **163**, 1539–1551 (2020).

9. Einbu, A. *et al.* Energy assessments of onboard CO2 capture from ship engines by MEA-based post combustion capture system with flue gas heat integration. *Int. J. Greenh. Gas Control* **113**, 103526 (2022).

10. DNV GL. Maritime Forecast to 2050. (2022).
